# EspH utilizes phosphoinositide and Rab binding domains to interact with plasma membrane infection sites and Rab GTPases*

**DOI:** 10.1080/19490976.2024.2400575

**Published:** 2024-09-23

**Authors:** Ipsita Nandi, Rachana Pattani Ramachandran, Deborah E. Shalev, Dina Schneidman-Duhovny, Raisa Shtuhin-Rahav, Naomi Melamed-Book, Efrat Zlotkin-Rivkin, Alexander Rouvinski, Ilan Rosenshine, Benjamin Aroeti

**Affiliations:** a Department of Biological Chemistry, The Alexander Silberman Institute of Life Sciences, The Hebrew University of Jerusalem, The Edmond J. Safra Campus – Givat Ram, Jerusalem, Israel; b Department of Cell and Developmental Biology, The Alexander Silberman Institute of Life Sciences, The Hebrew University of Jerusalem, The Edmond J. Safra Campus – Givat Ram, Jerusalem, Israel; c The Wolfson Centre for Applied Structural Biology, The Hebrew University of Jerusalem, The Edmond J. Safra Campus – Givat Ram, Jerusalem, Israel; d The Department of Pharmaceutical Engineering, Azrieli College of Engineering, Jerusalem, Israel; e The Rachel and Selim Benin School of Computer Science and Engineering, The Hebrew University of Jerusalem, Jerusalem, Israel; f Bioimaging Unit, Alexander Silberman Institute of Life Sciences, The Hebrew University of Jerusalem, Jerusalem, Israel; g Department of Microbiology and Molecular Genetics, Institute for Medical Research Israel-Canada, Faculty of Medicine, The Hebrew University-Hadassah Medical School, of Jerusalem, Jerusalem, Israel

**Keywords:** Enteropathogenic *e. coli*, type III secreted effectors, EspH, Rab GTPases, Rho GTPases, phosphoinositides (PIs), phosphoinositide binding domain (PBD) PI3K/Akt/mTORC1 signaling, lysosomal exocytosis, bacterial invasion, host-pathogen interactions

## Abstract

Enteropathogenic *E. coli* (EPEC) is a Gram-negative bacterial pathogen that causes persistent diarrhea. Upon attachment to the apical plasma membrane of the intestinal epithelium, the pathogen translocates virulence proteins called effectors into the infected cells. These effectors hijack numerous host processes for the pathogen’s benefit. Therefore, studying the mechanisms underlying their action is crucial for a better understanding of the disease. We show that translocated EspH interacts with multiple host Rab GTPases. AlphaFold predictions and site-directed mutagenesis identified glutamic acid and lysine at positions 37 and 41 as Rab interacting residues in EspH. Mutating these sites abolished the ability of EspH to inhibit Akt and mTORC1 signaling, lysosomal exocytosis, and bacterial invasion. Knocking out the endogenous Rab8a gene expression highlighted the involvement of Rab8a in Akt/mTORC1 signaling and lysosomal exocytosis. A phosphoinositide binding domain with a critical tyrosine was identified in EspH. Mutating the tyrosine abolished the localization of EspH at infection sites and its capacity to interact with the Rabs. Our data suggest novel EspH-dependent mechanisms that elicit immune signaling and membrane trafficking during EPEC infection.

## Introduction

The human gastrointestinal pathogens, enteropathogenic and enterohemorrhagic *Escherichia coli* (EPEC and EHEC), are food or water-borne bacterial pathogens that continue to pose a significant threat to human health worldwide.^1–3^ *Citrobacter rodentium* is a natural murine intestinal pathogen that shares a set of virulence factors with EPEC and EHEC.^4^ The type III secretion system (T3SS), which has a syringe-like molecular structure, is a major virulence factor of these pathogens.^5^ They utilize it to inject dozens of proteins, termed effectors, from the bacterial cytoplasm into the host enterocytes. The coordinated activity of these effectors in space and time subverts numerous host cell processes and organelles to support successful bacterial colonization of the intestinal mucosa.^6^ A prominent hallmark of the infection is the appearance of the so-called “attaching and effacing” (A/E) lesions in the mucosal tissue. A/E lesions are characterized by intimate microbial attachment to the apical cell plasma membrane of the epithelial cells, local elimination of brush-border microvilli, and the formation of a filamentous (F)-actin-rich pedestal-like structure on top of which the bacterium resides. Studies suggest that the A/E pedestal formation contributes to bacterial pathogenesis.^7^ Injected effectors also contribute to bacterial colonization and pathogenesis by hijacking and subverting host cell signaling and membrane trafficking pathways, targeting various organelles, and modulating cell death and innate immunity.^8–10^ One such effector is EspH.

EspH is essential for virulence and is a multifunctional effector.^11,12^ It regulates the actin cytoskeleton and pedestal formation,^13^ conferring Rho GTPase^14–18^ and MAP kinase^19^ inhibition, inducing host cell cytotoxicity,^15,17^ inhibiting bacterial invasion (phagocytosis),^12,16,17^ and increasing cell death due to mitochondrial fragmentation.^20^ Following translocation into the host cell, EspH localizes at plasma membrane infection sites.^15,17,19^ Despite this knowledge, the molecular mechanisms underlying EspH’s activities are not fully understood. In this context, EspH has been recently shown to interact with the host active Bcr-related (ABR) protein to suppress the host RhoGTPases, Rac1 and Cdc42.^17^ These interactions, mediated by the effector protein’s C-terminal 38 amino acid segment (domain), are required for EspH-mediated inhibition of bacterial invasion and filopodia formation at infection sites and EspH-evoked host cell cytotoxicity.^17^

Rab GTPases constitute the largest subfamily of the Ras-related GTPase superfamily, regulating multiple steps in vesicular trafficking in eukaryotic cells.^21–23^ Like all members of the GTPase superfamily, Rabs can bind GDP (guanosine-5’-diphosphate) in the “off-state” or GTP (guanosine-5’-triphosphate) in the “on-state.” Host proteins that regulate cycling between the two states are guanine nucleotide exchange factors (GEFs) that catalyze the GDP-GTP exchange reaction and GTPase activating proteins (GAPs) that facilitate the hydrolysis of bound GTP, thereby switching the Rabs on and off, respectively,^24^ and targeting them to distinct cellular compartments.^25^ Switching between the active and inactive forms is characterized by conformational changes of two critical regions within the GTPases, called switch I and II.^26^ The Rab proteins also interact with an additional group of host proteins called “effector” proteins. These proteins bind with high affinity to the switch regions of Rab’s active form,^27,28^ exerting specific cellular functions, some of which are overridden by intracellular bacterial pathogens.^29,30^

Studies have shown that human bacterial pathogens (e.g., *Salmonella enterica*, *Legionella pneumophila*, *Shigella flexneri*, *Listeria monocytogenes*) target Rab GTPases to establish a replication niche that enables their survival within the host cells.^30–32^ Moreover, studies have indicated that type III secreted effectors can target host Rab GTPases directly.^33–37^ *Salmonella’s* SopD and *Legionella’s* LidA are, perhaps, the best-studied examples, as crystal structures with their Rab cognates have been resolved.^38–40^ SopD binds and displays a GAP activity that inhibits Rab8a,^40,41^ and Rab10.^37^ Effector proteins can also act as GEFs. For example, the *Legionella’s* SidM (DrrA) effector recruits and activates Rab1 via its GEF domain. The same effector also acts as a GDP dissociation inhibitor displacement factor of Rab1.^42–44^ Effector proteins can modulate the activity of Rabs by covalently modifying them. For example, the AMPylation activity of the *Legionella* SidM/DrrA modifies Rab1 by covalently adding adenosine monophosphate (AMP) to avoid its recognition by GAPs.^45^ Another effector protein, SidD, acts as deAMPylase.^46,47^ Type III secreted effectors can also display protease activities on Rabs, such as *Salmonella*‘s GtgE effector, which acts as a Rab32 protease.^34^

Concerning functionality, the *salmonella* effector protein, SopD, has been shown to enhance or inhibit inflammatory responses by targeting Rab8a signaling.^40^ The *Legionella* LidA-Rab6a interactions were shown to be required for bacterial intracellular replication and growth.^48^ Notably, these effector-rab structure-function relationships have been characterized for invading bacterial pathogens. As far as we know, no knowledge exists about such relations for extracellular bacterial pathogens, including the A/E pathogens. Here, we show for the first time that translocated EspH functionally interacts with multiple active Rabs, including Rab8a, Rab10, Rab3a, and Rab12. We also show that EspH possesses a putative phosphoinositide-binding domain (PBD), which plays a pivotal role in EspH localization at plasma membrane infection sites, likely by mediating effector binding to phosphoinositides (PIs). The PBD is also vital for maintaining the EspH-Rab interactions.

## Materials and methods

### Bacterial strains, antibodies, plasmids, primers

Bacterial strains, antibodies, plasmids, primers, and reagents used in this study are listed in the supplementary Table S1.

### Cell culture and transfection of cells with plasmid DNA

HeLa and CaCo-2_BBe_ cells (semi-polarized) (see Table S1) were cultured, as previously described.^19^ Plasmids were transiently transfected into HeLa cells (~60% confluence) for 48 hrs unless otherwise indicated using the TransIT-X2 Transfection Reagent protocol.

### Construction of EspH mutants

The mutations were constructed on the pSA10-EspH_*wt*_-6×His-SBP encoding plasmid using PCR amplification of the vector and inserts and ligation using Gibson assembly. The vector was linearized using the 1F and 1 R oligonucleotides. The oligonucleotides 2F and 2 R or 3F and 3 R were used to mutate glutamic acid at position 37 to alanine or aspartic acid and generate the pSA10-EspH_*E37A*_ and pSA10-EspH_*E37D*_ (**E37A/D;** see Figure S1) encoding plasmids, respectively. Oligonucleotides 4F and 4 R or 5F and 5 R were used to mutate lysine at position 41 to alanine or arginine and generate the pSA10-EspH_*K41A*_ or pSA10-EspH_*K41R*_ (**K41A/R;** see Figure S1) encoding plasmids, respectively. Oligonucleotides 6F and 6 R were used to mutate lysine at position 106 to arginine and generate the pSA10-EspH_*K106R*_ plasmid. Oligonucleotides 7F and 7 R were used to mutate tyrosine at position 68 to alanine (**Y68A;** see Figure S1) and create the pSA10-EspH_*Y68A*_ plasmid. Nucleotide sequences of all constructs were confirmed by the Genomic Technologies Facility (https://www.bio.huji.ac.il/en/units_the_national_center_for_genomic_technologies.) using SANGER sequencing. All the EspH mutant plasmids were electroporated at 1.85 kV/25 µF/200 Ohm, using the BioRad electroporator (GENE PULSER II) into the EPEC-Δ*esp*H strains to generate the plasmid complemented bacterial strains.

### Bacterial pre-activation and cell infection

Before infection, the T3SS of bacterial strains was activated in plain high glucose Dulbecco’s Modified Eagle Medium (DMEM) for 3 hrs in the CO_2_ incubator (37°C, 5% CO_2_, 95% humidity), as described.^49^ The expression of EspH in EPEC-Δ*esp*H strains was induced by supplementing the activation medium with isopropyl-β-D-thiogalactopyranoside (IPTG; 0.05 mM for inducing EspH_*wt*_ expression, 0.1 mM for inducing the EspH mutant expression, and as indicated in the Figures) during the last 30 min of activation. Cell infection was performed with the pre-activated infection medium in the CO_2_ incubator at 37°C for the indicated times. Bacterial infection was performed at a multiplicity of infection (MOI) of ~100.

### SDS-PAGE and Immunoblotting

SDS-PAGE and immunoblotting (IB) were performed as described.^50^ Band intensity was measured using Fiji (NIH).

### Effector translocation assay

The effector translocation assay was performed as described.^49^ Approximately 2 × 10^5^ HeLa cells/well were seeded in a 6-well plate and cultured for 48 hrs until reaching ~70% confluence. Cells were then infected for 90 min at 37°C with the indicated EPEC strains, and IPTG was used to induce EspH expression. Following infection, cells were washed three times with ice-cold PBS and lysed in 60 µl of ice-cold lysis buffer [100 mM NaCl, 1 mM EDTA, 10 mM Tris-HCl, pH 7.4, 0.5% (vol/vol) NP-40] supplemented with protease and phosphatase inhibitors. Following 3 min incubation on ice, the lysate was pipetted up and down and then centrifuged (10,000 g, 4°C, 10 min). The supernatant (detergent-soluble) and pellet (detergent-insoluble) fractions were isolated. The pellet was resuspended in 60 µl of the lysis buffer. Twenty microliters of 4 × SDS-PAGE sample buffer were added to each lysis buffer containing fraction. The samples were then heated (95°C; 10 min) and analyzed by SDS-PAGE, followed by IB. The presence of EspH in the fractions was detected by anti-SBP antibodies. Anti-α-tubulin antibodies were used to assess the lysate protein load.

### Analyzing EspH-Rab interactions by co-precipitation (pulldown) assays

HeLa or Caco-2_BBe_ cells cultured on 15-cm plates (70% confluence) were infected for 90 minutes with pre-activated EPEC strains. Cells were washed three times with ice-cold PBS and lysed in ice-cold lysis buffer [50 mM Tris (pH 7.4), 150 mM NaCl, 0.5% NP-40] supplemented with protease and phosphatase inhibitors. Lysates were centrifuged (5,000 g, 15 min, 4 °C), and the Bradford reagent was used to determine the supernatants’ protein concentration. EspH was precipitated (P) from an equal amount (~5 mg) of cell lysates by incubation with 60 µl of Streptavidin (StAv) agarose beads (50% slurry pre-washed with lysis buffer) for 3 hrs at 4°C with end-to-end rotation. Beads were washed three times with lysis buffer by centrifugation (300 g, 2 min, 4 °C), dried using Hamilton’s syringe, and subjected to SDS-PAGE followed by IB. Pulled-down EspH and co-precipitated Rabs were detected using anti-SBP and appropriate anti-Rab or epitope-tagged antibodies.

### Fluorescence microscopy and colocalization analyses

Immunofluorescence labeling of permeabilized cells was performed as described.^51,52^

Cells were processed and imaged by an Olympus FV-1200 laser scanning confocal microscope equipped with a 60 × oil immersion objective (NA, 1.42), as described.^52^ Colocalization analyses using the intensity profile tool of Fiji (NIH) were performed, as described.^52^ Briefly, images for colocalization analyses were acquired under identical conditions. Colocalized labeling was scored when the fluorescence intensity co-peaked at a specific place along the line drawn to obtain a fluorescence intensity profile. Data are presented as percentages of colocalizing fluorescence intensities of the profiles. Notably, the anti-SBP antibodies recognized specifically the translocated EspH (see Figure S2). Infection with EPEC-Δ*espH* (not expressing EspH), or EPEC-*escV*/pEspH_*wt*_ (not translocating EspH) yielded low near background fluorescence levels compared to the high fluorescence intensity levels observed in cells infected with EPEC-Δ*espH*/pEspH_*wt*_ (translocating EspH) (Figure S2). Scale bars = 5 μm.

### The β-hexosaminidase activity (release) assay

Lysosomal exocytosis was evaluated using the β-hexosaminidase activity measurements in HeLa-infected cells, as described.^50^

### Bacterial invasion and filopodia formation measurements

Bacterial invasion and the induction of transient filopodia in infected HeLa cells were measured, as described.^17^

### Akt/mTORC1 activity measurements

HeLa or Caco-2_BBe_ cells cultured on 10-cm plates (70% confluence) were infected for 90 min with pre-activated EPEC. Cells were then washed three times with ice-cold PBS and lysed in ice-cold lysis buffer [50 mM Tris (pH 7.6), 150 mM NaCl, 10% glycerol, 1% Triton X-100, 1 mM EDTA, 2 mM MgCl_2_, 100 mM NaF, 200 μM NaVO_4_, 1 mM PMSF, 1 µM leupeptin and 1 µM aprotinin]. Lysates were centrifuged (10,000 g, 10 min, 4 °C), and the protein concentration of the supernatants was determined using the Bradford reagent. An equal amount of protein (~100 µg) was analyzed by SDS-PAGE, followed by IB. Active Akt and mTORC1 levels were evaluated by probing the blots with antibodies directed to phosphor (p) Akt (Ser473) or phosphor (p) 4E-BP1, respectively. The band intensities of phosphorylated proteins were first normalized to total (t) Akt or 4E-BP1 protein band intensities, then to the glyceraldehyde-3-phosphate dehydrogenase (GAPDH) protein band.

### Establishing a HeLa Rab8a knock-out (KO) cell line by a lentivirus-based CRISPR/Cas9 genome editing system

#### Lentivirus preparation

The gRNA sequences (8F’ and 8 R’; see Table S1) targeting the human Rab8a gene were cloned into lentiCRISPR V2 lentiviral vector (Table S1) as described by the Zhang laboratory (https://media.addgene.org/data/plasmids/52/52961/52961-attachment_B3xTwla0bkYD.pdf). To produce the lentiviruses, HEK293T/SF17 cells were seeded (3.8 × 10^6^ cells/plate) onto 10-cm plate and grown for 24 hrs in a CO_2_ incubator. Then, the cells were treated with 10 µl of 25 µM chloroquine and incubated in a CO_2_ at 37°C incubator for 5 hrs. Thereafter, the cells were transfected with a mixture of psPAX2 (10 µg), pMD2.G (6 µg), and either gRNA-containing lentiCRISPRV2 (10 µg) or empty lentiCRISPRV2 (10 µg) plasmids, using polyehyleneimine (PEI; DNA: PEI 1:3 w/w ratio), and incubated in the CO_2_ incubator for 16 hrs. Then, the transfection medium was replaced after 6 hrs with a complete DMEM lacking antibiotics to allow viral particle production and release for 48 or 96 hrs. The virus-containing medium was harvested, pooled, and centrifuged (500 × g, 10 min, 22 °C), and the supernatant was clarified by passing through a 0.45 μm filter unit. Viral particles were concentrated by gently mixing one volume of PEG8000 solution 32% w/v in PBS with 3 volumes of clarified supernatant and incubated for 30 min at 4°C, followed by centrifugation (1,500 × g 45 min 4°C). The viral pellet was dissolved in 12 ml of complete DMEM without antibiotics.

#### Cell infection and clone isolation

HeLa cells were seeded in a 6-well plate (1.5 × 10^5^ cells/well) and incubated for 24 hrs in the CO_2_ incubator before infection. The cells were then infected with 2 ml of the concentrated lentiviral preparation supplemented with 8 ug/ml polybrene and incubated for 24 hrs in the CO_2_ incubator. The cell infection treatment was repeated for three successive days. After the third infection, cells were washed with PBS and selected with complete DMEM containing puromycin (3 µg/ml) for 48 hrs. Cells were then cultured in 10-cm plates at 50 cells/plate in DMEM containing puromycin (3 µg/ml) and allowed to grow in the CO_2_ incubator for approximately ten days until single cell colonies were identified. Individual cell colonies were then picked by trypsinization and expanded for analysis of Rab8a expression by IB (Figure S3).

### Lactate dehydrogenase (LDH) cytotoxicity assay

HeLa cells (10,000 cells/well) were seeded on a 96-well plate and cultured for 48 hrs in a CO_2_ incubator until reaching ~70% confluence. Cells were infected with EPEC, and the LDH release assay was applied to the cell culture medium using the CytoTox 96® Non-Radioactive Cytotoxicity Assay (Promega). The cytotoxicity was calculated as follows: Cytotoxicity% = Experimental LDH Release (OD_490_)/Maximum LDH Release (OD_490_) [Percentage cytotoxicity=100×Experimental LDH Release  (OD490 Maximum LDH Release  (OD490], whereby ‘Experimental LDH Release’ denotes the LDH release into the medium bathing the cells; ‘Maximum LDH Release’ represents the LDH levels in cells lysed with 1X lysis solution provided by the kit.

### Statistical analysis

The GraphPad Prism v. 8.4.3 software was used for statistical analysis and graphing. A one-way ANOVA followed by Bonferroni’s multiple-comparison test was applied to determine the statistical significance. The significance is indicated by asterisks, as follows: ****p ≤ 0.0005; ***p > 0.0005; **p < 0.005; *p > 0.005; ns, non-significant *p* > 0.05. A p-value <0.05 indicates a statistically significant difference.

## Results

### Translocated EspH interacts with the human Rab GTPases Rab8a, 10, 3a, and 12

**Figure 1.**
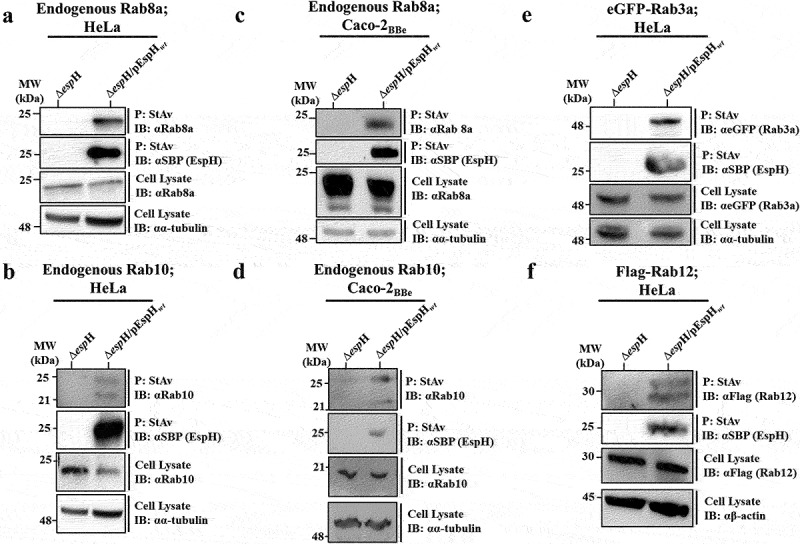
Rab8a, Rab10, Rab3a and Rab12 co-precipitate with translocated EspH_*wt*_.

Using co-precipitation followed by mass spectrometry and proteomics analysis, we have previously shown that EspH co-precipitated with multiple Rab GTPases, including Rab8a, Rab10, Rab3 (a and d), and Rab12, Rab1 (a and b) and Rab39a.^17^ It has been suggested that these Rabs interact with a region in EspH located upstream of the C-terminal 38-amino acid (aa) segment.^17^ The current study focused mainly on Rab8a (Figure 1a,c), Rab10 (Figure 1b,d), Rab3a [eGFP-Rab3a_*wt*_ (Figure 1e)], and, to some extent on Rab12 [Flag-Rab12_*wt*_ (Figure 1f)]. To confirm these findings by IB, HeLa (Figure 1a,b,e,f) or Caco-2_BBe_ (Figure 1c,d) cells were infected with an *espH* deleted EPEC strain (EPEC-Δ*espH*), used as a negative control, or with an EPEC-Δ*espH* complemented with a plasmid expressing wild-type (*wt*) EspH with six histidines (6 × His) and a streptavidin binding peptide (SBP) tags located at the C-terminus of the effector protein (EPEC-Δ*espH*/pEspH_*wt*_). Cells were lysed, and EspH was precipitated using Streptavidin (StAv) agarose beads. SDS-PAGE, followed by IB, was used to analyze precipitated EspH and co-precipitated Rabs. The results showed that the Rabs co-precipitated with EspH from the EPEC-Δ*espH*/pEspH_*wt*_ infected cells (Figure 1). Notably, the co-precipitated eGFP-Rab3a_*wt*_ (Figure 1e), Rab10 (Figure 1b,d) and Flag-Rab12_*wt*_ (Figure 1f) appeared as two protein bands, possibly as the result of Rab undergoing post-translational modifications.^53^

HeLa cells were infected with the indicated EPEC strains, lysed, and subjected to co-precipitation analyses, as described in Materials and Methods. SBP-tagged EspH_*wt*_ was precipitated (P) by streptavidin (StAv) beads, and anti-SBP antibodies were used to identify the precipitated EspH by IB. The co-precipitated endogenous Rab proteins were detected by IB, using antibodies directed against them (**panels a-d**) or anti-eGFP or anti-Flag tag antibodies in cases where epitope-tagged Rabs were ectopically expressed **(e and f**). The same antibodies were used to identify the Rabs in cell lysates. Anti-β-actin (αβ-actin) or anti-α-tubulin (αα-tubulin) antibodies were used for detecting protein loading. The endogenous Rab8a and Rab10 co-precipitation was examined in HeLa (a–b) and Caco-2_BBe_ (c–d) cells. The co-precipitation of the ectopically expresed eGFP-Rab3a and Flag-Rab12 with EspH was examined in HeLa cells (e-f). Representative gels from at least three independent experiments are shown.

Additionally, the endogenous Rab8a co-precipitated with EspH from EPEC-Δ*espH*/pEspH_*wt*_ and from cells infected with EPEC-Δ*espH* complemented with a plasmid expressing EspH whose C-terminal 38 aa ABR binding domain (Figure S1) was deleted (EPEC-Δ*espH*/pEspH_*∆130-168*_) (Figure S4). These results are consistent with our proteomics analyses,^17^ suggesting that the Rabs interact with the translocated EspH via a region located upstream to the C-terminal 38 aa segment.

#### EspH interacts with the active Rab forms

**Figure 2.**
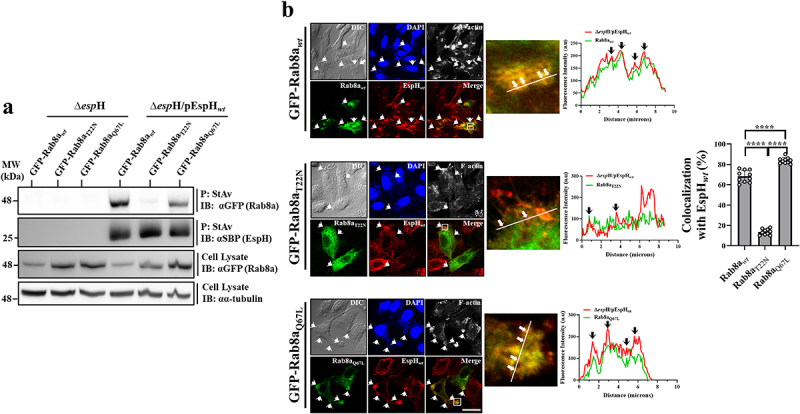
Translocated EspH_*wt*_ interacts with active Rab8a. Analysis by co-precipitation.

Using the same co-precipitation approach, we examined the capacity of translocated EspH_*wt*_ to co-precipitate the ectopically expressed GFP-Rab8a_*wt*_, eGFP-Rab3a_*wt*_ and eGFP-Rab10_*wt*_, the dominant negative (GDP-locked) GFP-Rab8a_T22N_, eGFP-Rab3a_T36N_ and eGFP-Rab10_T23N_ and the constitutively active (GTP-locked) GFP-Rab8a_Q67L_, eGFP-Rab3a_Q81L_ and eGFP-Rab10_Q58L_. The *wt*, the constitutively active but not the dominant negative Rabs, co-precipitated with EspH_*wt*_ from EPEC-Δ*espH*/pEspH_*wt*_ infected cells (Figure 2a and Figures. S5a and c). Confocal imaging confirmed these results, showing that translocated EspH_*wt*_ colocalized with the *wt* and the constitutively active but not with the dominant negative Rabs (Figure 2b and Figures S5b and d). These results suggest that EspH selectively binds active Rabs.

(a). HeLa cells ectopically expressing the wild-type (*wt*), T22N (GDP-locked), and Q67L (GTP-locked) GFP-tagged Rab8a were infected with EPEC-Δ*esp*H or EPEC-Δ*esp*H/pEspH_*wt*_ for 90 min at 37°C and subjected to co-precipitation experiments, as in Figure 1. A representative gel from three independent experiments is shown. **Colocalization analysis** (b). HeLa cells expressing the eGFP-tagged Rabs were infected with EPEC-Δ*esp*H/pEspH_*wt*_, fixed, permeabilized, and immunostained with anti-SBP antibodies to visualize EspH. Cells were also stained with DAPI (to visualize cell nuclei and bacterial microcolonies) and Phalloidin CF-647 (F-actin), and imaged by confocal microscopy. Representative images from three independent experiments are shown. The green (Rab) and the red (EspH) channels were merged. Arrows point toward infecting EPEC microcolonies. An enlargement of the boxed region in the merged image is shown, along with a line used to generate fluorescence intensity profiles of EspH and Rab. Arrows in the fluorescence intensity profiles point toward co-peaking fluorescence intensity signals. The bar graph depicts the percentage of colocalization between the Rabs and EspH derived from 10 intensity profiles. Results are mean ± SE. The results of parallel experiments involving eGFP-Rab3a and eGFP-Rab10 are shown in Figure S5a and b and Figure S5c and d, respectively.

#### AlphaFold calculated structures show a shared binding interface between EspH and the Rabs, where EspH residues E37 and K41 are implicated in Rab-binding

**Figure 3.**
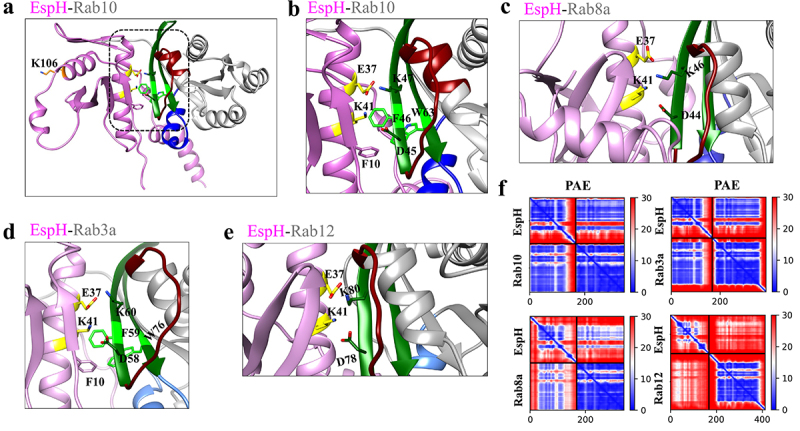
AlphaFold predicted structures for binding interfaces of EspH-Rabs.

AlphaFold-Multimer-v2.0 was used to calculate complexes of EspH (pink) and the different Rabs (gray) (Figure 3a–e). The predicted alignment error (PAE) showed high confidence in the binding regions of Rab3a and Rab10, with a somewhat reduced confidence in binding Rab8a and a significantly reduced confidence in binding Rab12 (Figure 3f), suggesting that the interactions of EspH with these Rabs are weak. In all calculated structures, residues E37 and K41 located in an α-helix of EspH interacted with corresponding Lys (K) and Asp (D) residues located in a β-strand of the interswitch region of the Rabs. Specifically, E37 and K41 of EspH with D45 and K47 residues of Rab10 (Figure 3b), D44 and K46 in Rab8a (Figure 3c), D58 and K60 in Rab3a (Figure 3d), and D78 and K80 in Rab12 (Figure 3e). F10 in EspH seems to interact with Rab10 or Rab3a (Figure 3b,d) via F46 and W63 in Rab10 and F59 and W76 in Rab3a. Interestingly, the aromatic F and W residues in the Rabs are among the three amino acids that make up the hydrophobic triad defined as F45, W62, and Y77 of human Rab8a, which interacts with the Legionella LidA effector.^38^ These additional interactions correlate with the increased confidence of binding of Rab10 and Rab3a with EspH, shown in the PAE plots (Figure 3f). Notably, the predicted Rab binding residues of EspH from different *E. coli* species are highly conserved, and as expected, are located upstream to the ABR binding C-terminal 38 aa domain (Figure S1).

The complexes of EspH-Rab10 (a-b), EspH-Rab8a (c), EspH-Rab3a (d), and EspH-Rab12 (e) were modeled using AlphaFold-Multimer-v2.0. The EspH (pink) and the Rab GTPase (gray) structures are depicted. The switch I, interswitch, and switch II Rab domains are shown in maroon, dark green, and navy blue, respectively. (a-b). **The EspH-Rab10 binding interface**. The Rab10-EspH complex is shown in **panel a**, and the interface area (dashed boxed) is enlarged in **panel b**, where the predicted interacting E37 and K41 of EspH (yellow) and K47 and D45 of Rab10 interswitch region (green) are shown. F10 (pink) of EspH interacts with F46 (bright green) and W63 (bright green) of Rab10. The non-interacting K106 of EspH (orange) is also indicated (**panel a**). (c). **The EspH and Rab8a binding interface**. (d). **The EspH-Rab3a binding interface**. (e). **The EspH-Rab12 binding interface**. The interacting E37 and K41 residues of EspH (yellow) respectively interact with K46 and D44 in Rab8A (c), with K60 and D58 in Rab3a (d), and with K80 and D78 in Rab12 (e), all located in the interswitch region (green). F10 of EspH (pink) interacts with F59 and W76 in Rab3a (d). (f). **P****redicted** **A****ligned** **E****rror (PAE) plots for EspH and the denoted Rab proteins**. The PAE matrices were calculated for each EspH-Rab complex using the AlphaFold program. In these matrices, both axes show the position of the indicated residues of both proteins, starting with EspH, consecutively numbered. The score presents the calculated error of the predicted distance for each pair of residues color-coded from blue (0 angstroms) to red (30 angstroms), as shown in the right bar, where a low predicted error, blue, indicates higher certainty regarding the relative position of the two amino acids. The diagonal blue shows amino acids that are sequential in the primary sequence and are also adjacent in space. Blue coding between EspH and the Rabs in the off-horizontal region indicates high certainty in the regions of intermolecular interaction.

#### Residues E37 and K41 of EspH are crucial for Rab binding

**Figure 4.**
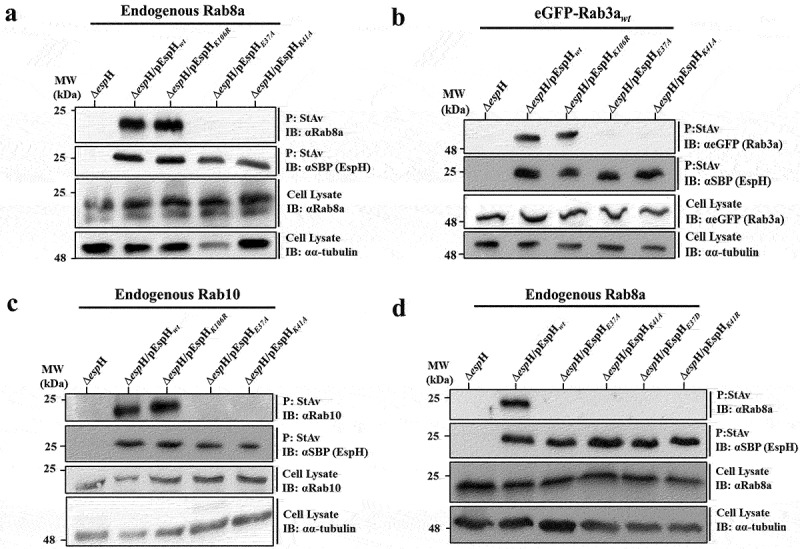
The predicted Rab binding residues in EspH are critical for EspH-Rab interactions and are highly susceptible to mutagenesis.

We performed site-directed mutagenesis to explore the significance of E37 and K41 of EspH in Rab binding, whereby the indicated residues were individually mutated to alanine (A) (Figure S1). EPEC-Δ*espH* mutant strains complemented with plasmids encoding the EspH mutant EspH_*E37A*_ (EPEC-Δ*espH*/pEspH_*E37A*_) or EspH_*K41A*_ (EPEC-Δ*espH*/pEspH_*K41A*_) were generated, and their ability to express and translocate EspH was confirmed (Figure S6a and b). HeLa cells were infected with these bacterial strains and subjected to the Rab co-precipitation approach. The results show that EspH_*E37A*_ and EspH_*K41A*_ failed to co-precipitate endogenous Rab8a (Figure 4a), eGFP-Rab3a_*wt*_ (Figure 4b), and the endogenous Rab10 (Figure 4c). Similar results were obtained with the endogenous Rab8a in Caco-2_BBe_ infected cells (Figure S7).

HeLa cells were infected for 90 min at 37°C with the indicated EPEC strains. The capacity of the endogenous Rab8a (a and d), eGFP-Rab3a_*wt*_ (b), and endogenous Rab10 (c) to co-precipitate with EspH was examined as described in Materials and Methods, and Figure 1. Representative gels from three independent experiments are shown.

Next, we explored whether conservative mutations of E37 and K41 also affected Rab binding. For this purpose, EPEC-Δ*espH* complemented with EspH_E37D_, or EspH_K41R_ encoding plasmids were generated (Figure S1). The mutant effector translocation into HeLa cells was confirmed (Figure S6d and e). Interestingly, these EspH mutants also failed to co-precipitate the endogenous Rab8a (Figure 4d). Conversely, mutating a remotely located K106 to arginine (K106R; see Figure S1), whose expression and translocation into the host cells have been confirmed (Figure S6c), did not detectably impact EspH’s ability to co-precipitate the Rabs (Figure 4a–c). Consistent with the co-precipitation data, colocalization analysis of translocated EspH_*K106R*_, EspH_*E37A*,_ and EspH_*K41A*_ with GFP-Rab8a, eGFP-Rab10, or eGFP-Rab3a, expressed in HeLa cells showed that while EspH_*K106R*_ colocalized significantly with the Rabs, the other two mutants did not (Figure S8a–d). These results support the understanding that residues E37 and K41 of EspH are critical for mediating the EspH-Rab interactions.

#### EspH-Rab interactions are functionally relevant

Next, we investigated the impact of EspH-Rab interactions on the infected cells, focusing on previously suggested roles for these Rabs. For example, Rab8a and Rab10 have been implicated in regulating class I phosphoinositide 3-kinase (PI3K)/mammalian target of rapamycin complex 1 and 2 (mTORC1/C2) signaling.^54–62^ Therefore, EspH-Rab8a interactions may affect the PI3K/Akt/mTOR signaling pathways. To address this hypothesis, we have initially tested whether translocated EspH_*wt*_ affects the levels of active Akt and mTORC1 by IB using antibodies directed against phosphorylated Ser473 of active Akt [α-pAkt (Ser473)], or against phosphorylated Thr37/46 of active 4EBP1 [α-p4EBP1 (Thr37/46)]. In these experiments, HeLa cells were infected, or not (uninfected), with EPEC-*escV*, EPEC_*wt*_, EPEC-Δ*espH*, EPEC-Δ*espH*/pEspH_*wt*_. Cells were lysed, and cell lysates were subjected to SDS-PAGE followed by IB analyses using the indicated antibodies. Cell infection with EPEC-*escV* increased pAkt levels compared to uninfected cells (Figure S9), a phenomenon that the bacterial lipopolysaccharide could contribute.^60^ Infection with EPEC_*wt*_ diminished the pAkt and p4EBP1 levels compared to EPEC-*escV* infected cells. Interestingly, infection with EPEC-Δ*espH* unaffected those levels, whereas infection with EPEC-Δ*espH*/pEspH_*wt*_ caused a significant reduction in both pAkt and p4EBP1 (Figure S9). These data argue that translocated EspH_*wt*_ inhibits the PI3K/Akt/mTORC1 signaling.

**Figure 5.**
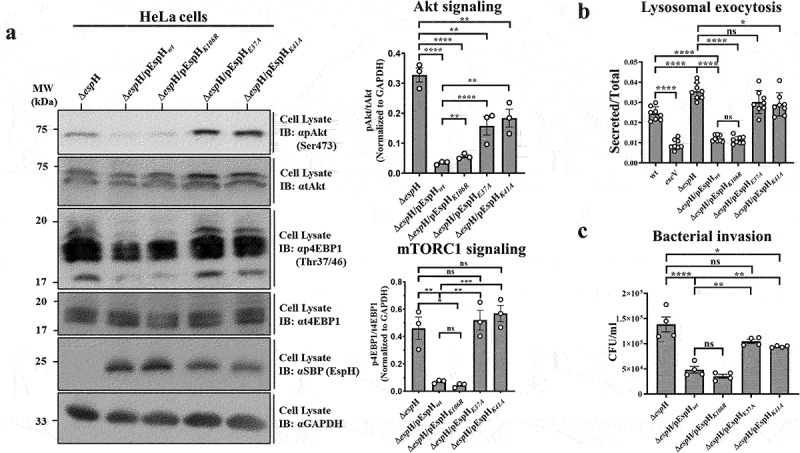
The Rab binding residues in EspH are critical for exerting Rab-related functions.

To explore the role of EspH-Rab interactions, HeLa cells were infected with EPEC-Δ*espH*, EPEC-Δ*espH*/pEspH_*wt*_, EPEC-Δ*espH*/pEespH_*K106R*_, EPEC-Δ*espH*/pEspH_*E37A*_ and EPEC-Δ*espH*/pEspH_*K41A*_ and the Akt and mTORC1 activity level was evaluated as described above. Cell infection with EPEC-Δ*espH*/pEspH_*wt*_ or EPEC-Δ*espH*/pEspH_*K106R*_ resulted in a significant diminishment of pAkt(Ser473) and p4EBP1 (Thr37/46) levels compared to EPEC-Δ*espH* infected cells (Figure 5a). In contrast, the levels of pAkt (Ser473) and p4EBP1 (Thr37/46) in cells infected with EPEC-Δ*espH*/pEspH_*E37A*_, or EPEC-Δ*espH*/pEspH_*K41A*_ were comparable to those detected in EPEC-Δ*espH* infected cells (Figure 5a). Similar results were obtained in Caco-2_BBe_ infected cells (Figure S10). These results indicate that EspH inhibits the pI3K/Akt/mTORC1 signaling pathway, possibly through inhibiting Rab8a and Rab10.

HeLa cells were infected with the indicated EPEC strains for 90 min at 37°C, and the effects on Akt and mTORC1 signaling (a), lysosome exocytosis (b), and bacterial invasion (c) were measured, as described in Materials and Methods. All experiments were repeated at least three times. Representative gels (**panel a, left**) from three independent experiments and their quantification (**panel a, right**) are shown. Results are mean ±SE.

Rab3a and Rab10 have been implicated in regulating lysosomal exocytosis.^63–65^ We have recently shown that EPEC infection stimulates lysosomal exocytosis in a T3SS-dependent manner, involving the death-promoting effectors Tir, EspF, and Map.^50^ The observation that EspH interacts with Rab3a and Rab10 (Figures 1,3,4, S5a–d) prompted the hypothesis that the effector modulates lysosomal exocytosis. To test this, cells were infected with EPEC-*wt*, or EPEC-*escV*, or EPEC-Δ*espH*/pEspH_*wt*_, or EPEC-Δ*espH*/pEspH_*K106R*_, or EPEC-Δ*espH*/pEspH_*E37A*_, or EPEC-Δ*espH*/pEspH_*K41A*_. The β-hexosaminidase activity (release) assay was applied to measure lysosomal exocytosis.^50^ Infection with EPEC-*escV* showed reduced lysosomal exocytosis compared to EPEC-*wt*-infected cells, confirming the involvement of the T3SS in the process (Figure 5b). EPEC-Δ*espH* infection resulted in slightly higher lysosomal exocytosis levels than in EPEC-*wt* infected cells (Figure 5b). Infection with EPEC-Δ*espH*/pEspH_*wt*_ or EPEC-Δ*espH*/pEspH_*K106R*_ caused a significant reduction in these levels (Figure 5b), suggesting that injected EspH inhibits lysosomal exocytosis. In contrast, infection with EPEC strains expressing the Rab-interaction deficient mutants (EspH_*E37A*_ and EspH_*K41A*_) showed higher lysosomal exocytosis levels (Figure 5b). These data suggest that translocated EspH inhibits lysosomal exocytosis, possibly through binding and modulating the activity of Rab3a or Rab10.

Studies on *Salmonella* showed that inhibiting Rab10 by the SopD effector promoted Dynamin-2 recruitment and plasma membrane scission during bacterial invasion.^37^ Studies have also shown that EspH inhibits EPEC invasion by inhibiting CDC42 and Rac1 GTPases.^16,17^ However, these observations do not exclude the existence of other mechanisms, such as the interactions of EspH with host Rabs. Infection with EPEC-Δ*espH*/pEspH_*wt*_, or EPEC-Δ*espH*/pEspH_*K106R*_ displayed significantly reduced invasion levels compared to EPEC-Δ*espH*. In contrast, infection with EPEC-Δ*espH*/pEspH_*E37A*_ or EPEC-Δ*espH*/pEspH_*K41A*_ showed higher invasion levels, comparable to those displayed by EPEC-Δ*espH* infected cells (Figure 5c). These data support the hypothesis that EspH inhibits bacterial invasion by binding and modulating the activity of Rabs, possibly Rab10.

#### Effects of EspH on Rab8a deficient cells

**Figure 6.**
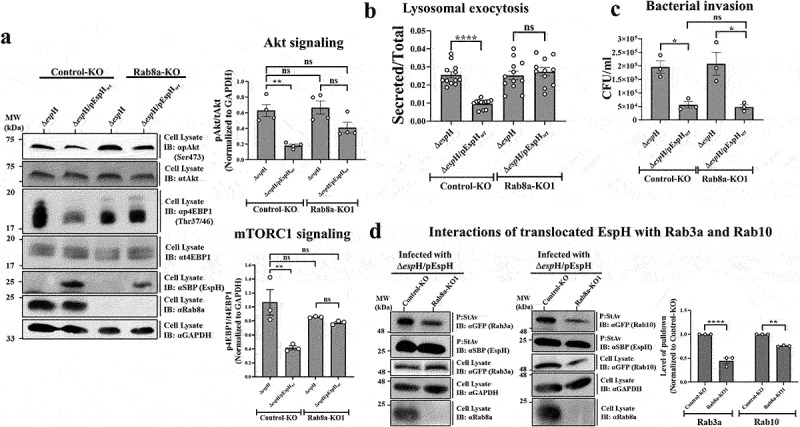
Interactions of EspH with Rab8a are essential for eliciting the AkT/mTORC1 signaling, lysosomal exocytosis, and the interactions with Rab3a.

Given the vital role of Rab8a in PI3K/Akt/mTORC1 signaling as a mechanism that limits innate immune responses,^60,62^ we studied whether the EspH-mediated inhibition of these signaling pathways (Figure 5a) depends on Rab8a. For this purpose, a lentiviral vector-based CRISPR/Cas9 genome editing system was used to generate HeLa cell lines deficient in Rab8a expression [Rab8a-knock-out (KO) cells, Figure S3]. HeLa cells transduced with empty lentiviral vector and, therefore, not hampered Rab8a expression were used as controls in these experiments (Control-KO cells). To test the effects of translocated EspH, data obtained in EPEC-Δ*espH*/pEspH_*wt*_ infected cells were compared to those of EPEC-Δ*espH* infected cells. While a significant reduction in the pAkt and p4EBP1 levels was observed in the Control-KO cells infected with EPEC-Δ*espH*/pEspH_*wt*_, a minor insignificant reduction was seen in the Rab8a-KO1 cells (Figure 6a). These results agree with the view that EspH-Rab8a interactions play a role in the inhibition of PI3K/Akt/mTORC1 signaling. In addition, our results show that the inhibition of lysosomal exocytosis is significantly reduced in the Control-KO but not in the Rab8a-KO1 EPEC-Δ*espH*/pEspH_*wt*_ infected cells (Figure 6b), suggesting that EspH-Rab8a interactions could play a vital role in lysosomal exocytosis inhibition. However, the inhibition of bacterial invasion by translocated EspH was reduced to similar levels in the Control-KO and Rab8a-KO1 cells (Figure 6c), indicating that EspH-Rab8a interactions are not involved in bacterial invasion. Co-precipitation followed by IB analysis showed that eGFP-Rab3a and eGFP-Rab10 co-precipitated with translocated EspH from the Rab8a and Control-KO cells, albeit with different efficacies. The Rab3a and Rab10 co-precipitated at lower levels in the Rab8a KO1 cells compared to the Control-KO cells (Figure 6d). These results suggest that the interactions between EspH and Rab8a could play a role in maintaining optimal interactions with other Rabs.

Control-KO and Rab8a-KO1 HeLa cells (Figure S3) were infected with the indicated EPEC strains for 90 min at 37°C, and the effects on Akt and mTORC1 signaling (a), lysosome exocytosis (b) and bacterial invasion (c), were measured, as described in Materials and Methods and Figure 5. (d) Interactions of translocated EspH with Rab3a and Rab10. Control-KO and Rab8a-KO1 HeLa cells were transfected with eGFP-Rab3a and eGFP-Rab10 encoding plasmids for 48 hrs and then infected for 90 min with the indicated EPEC strains. Co-precipitation experiments were performed, as in Figure 1. Precipitated EspH and co-precipitated Rabs were detected with anti-SBP and anti-GFP antibodies, respectively. Rab levels in cell lysates were detected with anti-GFP antibodies. Anti-GAPDH antibodies were used to assess the protein load. The level of the co-precipitated (‘pulldown’) Rabs was calculated by measuring the intensity of the co-precipitated protein band normalized to the intensity of the protein band detected in the cell lysate and the intensity of the GAPDH band. The values obtained were further normalized to the Control-KO levels. All experiments were repeated at least three times. Representative gels from at least three independent experiments are shown. Results are mean ±SE.

#### EspH-Rab interactions do not play a role in stimulating host cytotoxicity and filopodia repression

**Figure 7.**
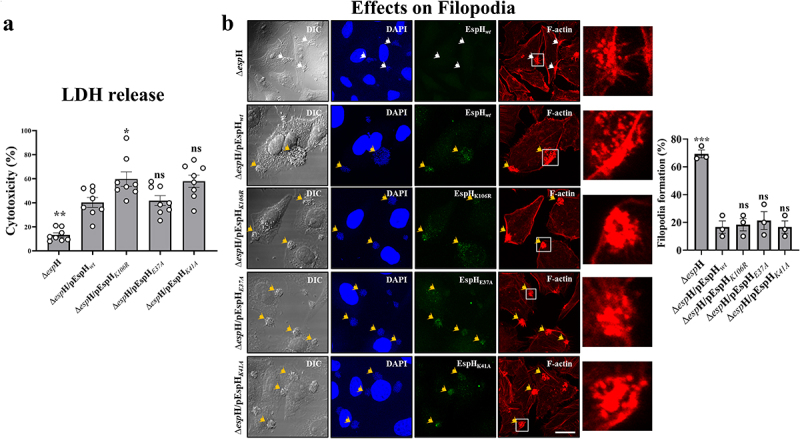
The effects of mutations in EspH used to study the interactions with Rab GTPases on host cell cytotoxicity and filopodia formation.

Studies have shown that translocated EspH induces host cell cytotoxicity and inhibits transient filopodia formation, functions attributed to Rho GTPase inhibition.^17^ Infection of HeLa cells with EPEC-Δ*espH*/pEspH_*E37A*_ or EPEC-Δ*espH*/pEspH_*K41A*_ did not affect host cell cytotoxicity (Figure 7a) or filopodia repression (Figure 7b) compared to EspH_*wt*_ infected cells, suggesting that Rab binding by EspH is not involved in these processes. These results further signify the existence of two distinct functional motifs in EspH: one is the C-terminal 38aa segment that binds ABR to downregulate Rho GTPases, inducing cell cytotoxicity and repressing filopodia formation,^17^ and the other is the Rab binding motif located upstream of this segment, involved in modulating Rab GTPases and their role in Akt/mTORC1 signaling, lysosomal exocytosis and bacterial invasion.

HeLa cells were infected for 90 or 15 min with the indicated EPEC strains, and the impact on cell cytotoxicity using the LDH release assay (a) or filopodia formation by confocal cell imaging (b), respectively, was measured as described in Materials and Methods. For cell imaging, EspH was immunostained by anti-SBP antibodies, host cell nuclei and bacterial microcolonies were visualized by DAPI staining, and F-actin was observed by Phalloidin Texas Red staining. Representative images from three independent experiments are shown. Results are mean ± SE. Statistical significance tests were performed versus the EPEC-∆*esp*H/EspH_*wt*_ infected cells.

#### A PBD of EspH mediates effector localization at plasma membrane infection sites and interactions with Rab8a

**Figure 8.**
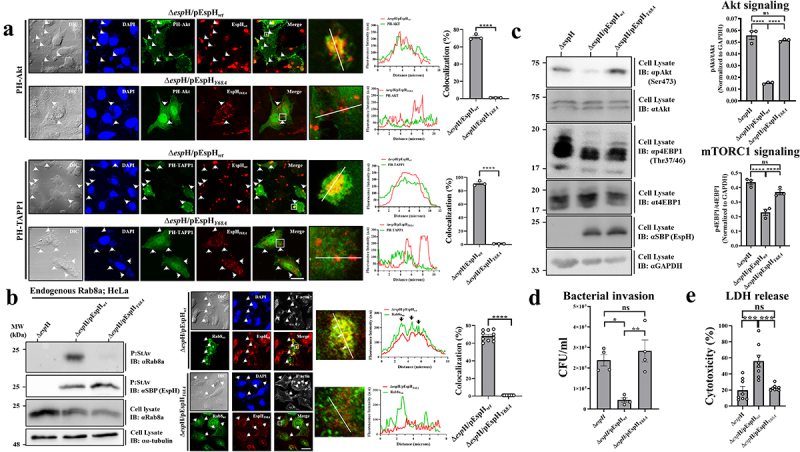
The EspH PBD is critical for EspH localization at infection sites, PI clustering, and interactions with Rab8a.

A common conserved GKxYx_n_F PBD with a critical tyrosine has been identified in type III-secreted effectors, mediating high-affinity interactions with PI-enriched membrane platforms.^66^ Interestingly, we identified the conserved domain in EspH expressed by several pathogenic *E. coli* species, including EPEC, and thus mutated its conserved tyrosine at position 68 to alanine (Y68A; Figure S1). The expression and translocation of EspH_*Y68A*_ into HeLa cells infected with EPEC-Δ*espH*/pEspH_*Y68A*_ was confirmed (Figure S6f). HeLa cells expressing eGFP-PH-Akt [a reporter of PI(3,4)P_2_ and PI(3,4,5)P_3_] or eGFP-PH-TAPP1 [a reporter of PI(3,4)P_2_]^67^ were infected with EPEC-Δ*espH*/pEspH_*wt*_ or EPEC-Δ*espH*/pEspH_*Y68A*_. Confocal imaging showed that translocated EspH colocalized extensively with each PI reporter in large patches at the plasma membrane infection sites. The translocated EspH_*Y68A*_ neither clustered nor colocalized with the PI sensors at the infection sites (Figure 8a). These results may agree with previous reports, suggesting the existence of PI-enriched membrane platforms at infection sites^68–70^ with which EspH interacts via its PBD. These interactions may promote EspH preferential localization and PI clustering at these sites.

(a). **PI clustering at infection sites**. HeLa cells were transfected with eGFP-PH-Akt (upper) or eGFP-PH-TAPP1 encoding plasmids (lower). Eighteen hours post-transfection, cells were infected with EPEC-Δ*espH*/pEspH_*wt*_ or EPEC-Δ*espH*/pEspH_*Y68A*_ strains for 30 min at 37°C. Cells were fixed, permeabilized, and immunostained with anti-SBP antibodies to visualize EspH (red). Cells were then stained with DAPI (blue) to visualize host nuclei and adhered bacterial microcolonies (indicated with arrowheads) and analyzed by confocal microscopy. The colocalization analysis was performed as described in Figure 2b. The bar graph depicts the percentage of colocalization derived from 3 intensity profiles. (b) **Interactions with Rab8a;*Analysis by co-precipitation (left)***. HeLa cells were infected for 90 min with the indicated EPEC strains, and co-precipitation experiments detecting the endogenous Rab8a were performed, as in Figure 1. **Analysis by colocalization (right)**. HeLa cells were transfected with a GFP-Rab8a_*wt*_ encoding plasmid and infected with the indicated EspH-expressing EPEC strains. Colocalization analyses were performed, as described in Materials and Methods and Figure 2b. (c) **Effects on Akt/mTORC1 activities**. HeLa cells were infected for 90 min with the indicated EPEC strains, and the activity assay was performed as described in Materials and Methods and Figure 5. (d) **Effects on bacterial invasion**. HeLa cells were infected for 90 min at 37°C with the indicated EPEC strains, and the invasion assay was performed as described in Materials and Methods and Figure 5c. (e) **Effects on host cytotoxicity**. HeLa cells were infected with the indicated strains for 90 min at 37°C, and the LDH release assay was used to evaluate the impact on cell cytotoxicity. All experiments were repeated at least three times. Representative gels are shown. Results are mean ±SE.

Next, we examined whether the PBD is essential for the EspH-Rab8a interactions. HeLa cells infected with EPEC-Δ*espH* (a negative control) EPEC-Δ*espH*/pEspH_*wt*_ or EPEC-Δ*espH*/pEspH_*Y68A*_, were subjected to the Rab co-precipitation method. The endogenous Rab8a co-precipitated with EspH_*wt*_ but not EspH_*Y68A*_ (Figure 8b, left). Confocal imaging showed significant colocalization between GFP-Rab8a and translocated EspH_*wt*_, but not EspH_*Y68A*_, in large clusters at infection sites (Figure 8b, right). Notably, the Alphafold predicted structure of EspH-Rab8a suggested that Y68 of the EspH PBD is distinct from the Rab binding interface (Figure S11). While infection with EPEC-Δ*espH*/pEspH_*wt*_ showed a significant reduction of pAkt and p4EBP1 levels compared to EPEC-Δ*espH*, cells infected with EPEC-Δ*espH*/pEspH_*Y68A*_ displayed an impaired ability to dephosphorylate Akt and 4EBP1 (Figure 8c). While infection with EPEC-Δ*espH*/pEspH_*wt*_ caused significant inhibition of bacterial invasion compared to EPEC-Δ*espH* infected cells, infection with EPEC-Δ*espH*/pEspH_*Y68A*_ did not yield an effect (Figure 8d). Unlike translocated EspH_*wt*_ which, as previously demonstrated,^15,17^ induced cytotoxicity, translocated EspH_*Y68A*_ had no impact on host cytotoxicity (Figure 8e). These data argue that the PBD of EspH is significant for its interactions with host Rab8a. They also indicate that the interactions are important for exerting EspH-dependent suppression of Akt, mTORC1, bacterial invasion into the host cells, and induction of host cytotoxicity. Thus, the interactions of EspH with plasma membrane PIs are vital for exerting all its reported functions.

## Discussion

EspH, an effector protein known for its ability to disrupt the host cell actin cytoskeleton by inhibiting Rho GTPases,^14–19,71^ is shown here to interact with several Rab GTPases, including Rab8a, Rab10, Rab3a, and Rab12 (Figures 1–4). In this regard, EspH joins a list of bacterial effector proteins shown to bind multiple host Rabs.^33–40^ The ability of EspH to bind these Rabs at different time points of infection and precipitate them from cell lysates (Figure S12) suggests that the binding is firm and occurs even at an early infection phase, an idea consistent with reports for other effector-Rab interactions.^32,38^ The mechanism by which EspH affects the host Rabs remains unknown. However, it would be conceivable to assume that it inhibits normal signaling pathways by binding the active Rab forms (Figure 2 and Figure S5). It would also be reasonable to hypothesize that EspH-mediated inhibition of Akt/mTORC1 signaling by binding active Rab8a (Figure 6a) causes the inhibition of downstream cytokine secretion and inflammatory responses, as described earlier in the case of lipopolysaccharide-treated macrophages^60,62^ and for the *Salmonella* SopD effector protein.^40^

We have recently shown that lysosomal exocytosis is linked to the translocation of death-promoting effectors, e.g., Tir, EspF, and Map.^50^ Here, we show that translocated EspH inhibited lysosomal exocytosis in a Rab8a-dependent fashion (Figure 6b). mTORC1 signaling has been tightly linked to lysosomal positioning and trafficking.^72–74^ Hence, the downregulation of mTORC1 activity by EspH-Rab8a interactions (Figure 6a) may play a role in lysosomal exocytosis inhibition (Figure 6b). The efficiency of Rab3a to co-precipitate with EspH is reduced in the absence of Rab8a (Figure 6d), suggesting that the capacity of the effector to interact with Rab3a depends on Rab8a binding. As Rab3a has also been proposed to modulate lysosomal exocytosis,^63,64^ the reduced capacity of EspH to inhibit lysosomal exocytosis in the Rab8a-deficient cells may be contributed by reduced Rab3a binding. EspH induces cell cytotoxicity, imposing cell rounding and plasma membrane damage.^17^ Lysosomal exocytosis regulated by Rab10 and Rab3a has been implicated in membrane repair.^64^ The potential blockade of host membrane repair by EspH may represent a novel type of cell stress imposed by the effector.

AlphaFold identified residues in an α-helix of EspH (E37 and K41) that bind the K and D residues in the β-strand interswitch region of all the indicated Rabs, suggesting this is a common characteristic in Rab binding (Figure 3). Crystallographic studies have found similar effector-Rab interactions: e.g., α-helices in *Legionella* LidA that bind the two switches and the interswitch of Rab1 via polar aa and salt bridges.^39^ The structure of the complex (PDBid 3SFV) shows interactions between K49 and D47 on a β-strand of Rab1 to the corresponding D235 and K239 on LidA, similar to the E37 to K and K41 to D of EspH-Rab interactions. Similarly, the *Salmonella* SopD effector displays a large interaction interface with Rab8a, including experimentally identified E293 and K285 in an α-helix in the effector protein that interacts with K58 and Q60, respectively, in the interswitch β-strand of Rab8a.^40^

A hydrophobic interaction was seen between the hydrophobic triad (F45, Y77, and W62) in human Rab8a and LidA.^38^ We also found aromatic interactions between F46 and W63 of the hydrophobic triad of Rab10 and F59 and W76 of Rab3a, with F10 of EspH (Figure 3b,d). These interactions may contribute to stronger binding, which may be why the PAE confidence is higher for these complexes in the AlphaFold structures (Figure 3f). In conclusion, the Rab-effector structural interface, exemplified in the case of EspH-Rab binding and found in other bacterial effector-Rab interfaces, may reflect a common mode of binding that enables the functional manipulation of multiple Rabs by these effectors and contributes to pathogenesis.

Our data indicate that plasma membrane PIs play a role in enabling the EspH-Rab interactions (Figure 8). PI sensors and translocated EspH_*wt*_ clustered significantly at the infection sites. The clustering effect was markedly reduced in the presence of EspH_*Y68A*_ (Figure 8a). Interestingly, Tir has been suggested to buildup of PI(3,4)P_2_ and PI(3,4,5)P_3_ enriched platforms at plasma membrane infection sites.^68–70^ EspH may further augment this effect by binding and clustering host PIs via its PBD. Plasma membrane PI domains have been implicated in the recruitment and regulation of Rab and Rho GTPases spatiotemporal signaling.^75–77^ Therefore, the EspH-PI interactions at bacterial infection sites may represent another mechanism by which the effector protein impacts the structure and function of host Rab and Rho GTPases.

As an extracellular bacterial pathogen, EPEC elicits mechanisms that block its invasion (phagocytosis) into the host cells, enabling it to remain extracellular.^78^ These mechanisms involve the inhibition of Rho GTPases by EspH,^16,17^ EspG-mediated counteracting of the WAVE regulatory complex,^79^ and inhibition of PI3K-dependent signaling pathways.^80^ Here, we propose another anti-phagocytic mechanism involving EspH binding to host Rabs (Figure 5c), possibly by binding Rab10.^37^ This hypothesis will be further addressed by knocking out or down the expression of Rab10 in the host cells.

In summary, our studies suggest that the effector protein EspH of A/E pathogens contains PI, ABR, and Rab GTPase binding domains. EspH utilizes a PBD to confine its localization at plasma membrane infection sites. These interactions facilitate the binding and downregulation of the activity of host Rho (through binding ABR) and Rab GTPases to disrupt the actin cytoskeleton, lysosomal trafficking, and immune signaling pathways, thereby contributing to bacterial pathogenesis.^11^

## Supplementary Material

Supplemental Material

## Data Availability

The data that support the findings of this study are available from the corresponding author, BA, upon reasonable request.
