# Supplementary material for: EspH utilizes phosphoinositide and Rab binding domains to interact with plasma membrane infection sites and Rab GTPases*
Source: Gut Microbes. 2024 Sep 23;16(1):2400575. doi: 10.1080/19490976.2024.2400575 (PMC11421376; doi:10.1080/19490976.2024.2400575)
Supplement: Supplemental Material [file KGMI_A_2400575_SM0660.zip › Nandi_et_al_Supplementary_Figs_R1_changes_after_proof_.pptx]

## Slide 1
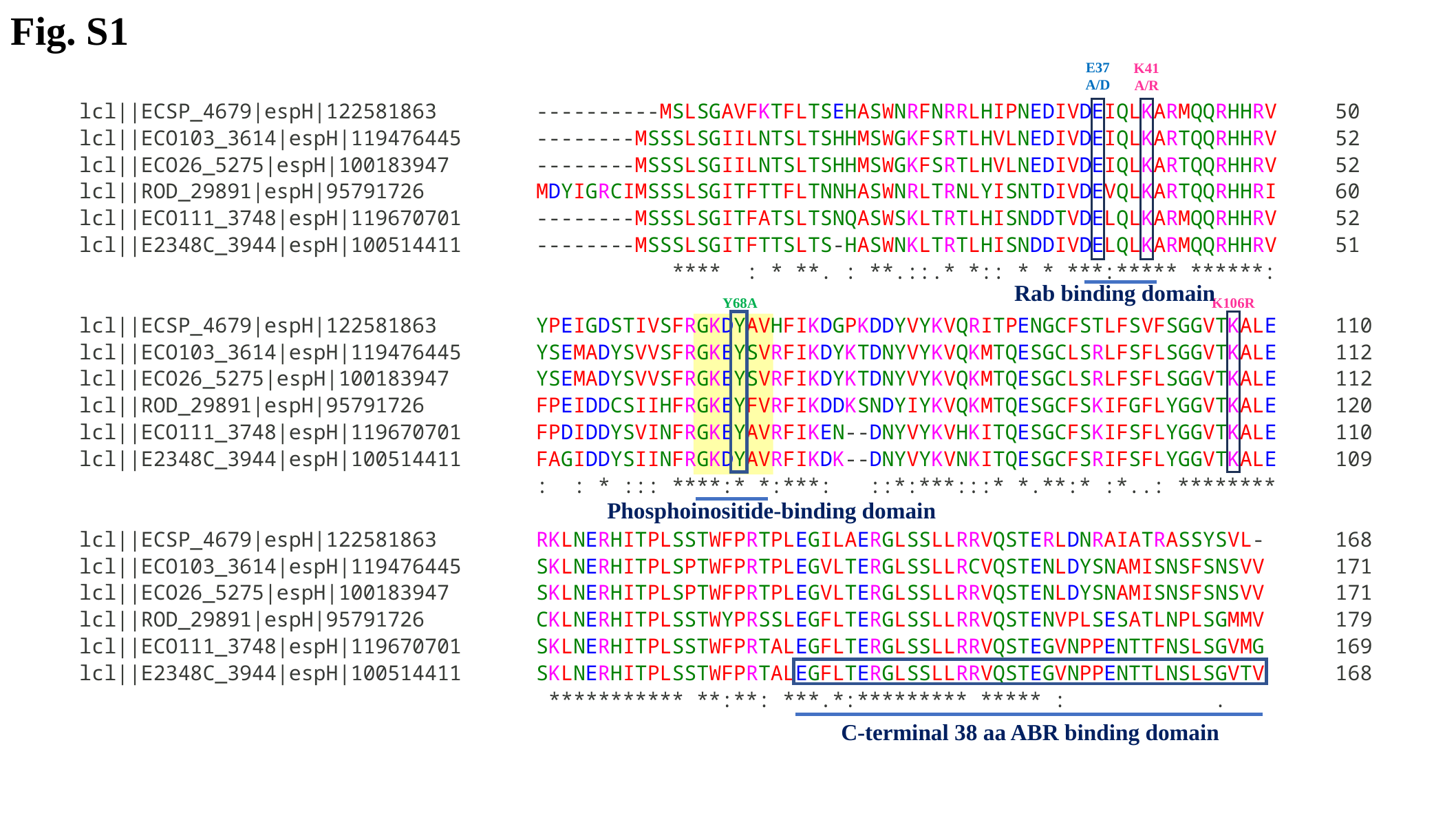

Fig. S1
E37
A/D
K41
A/R
K106R
Rab binding domain
Y68A
Phosphoinositide-binding domain
C-terminal 38 aa ABR binding domain

## Slide 2
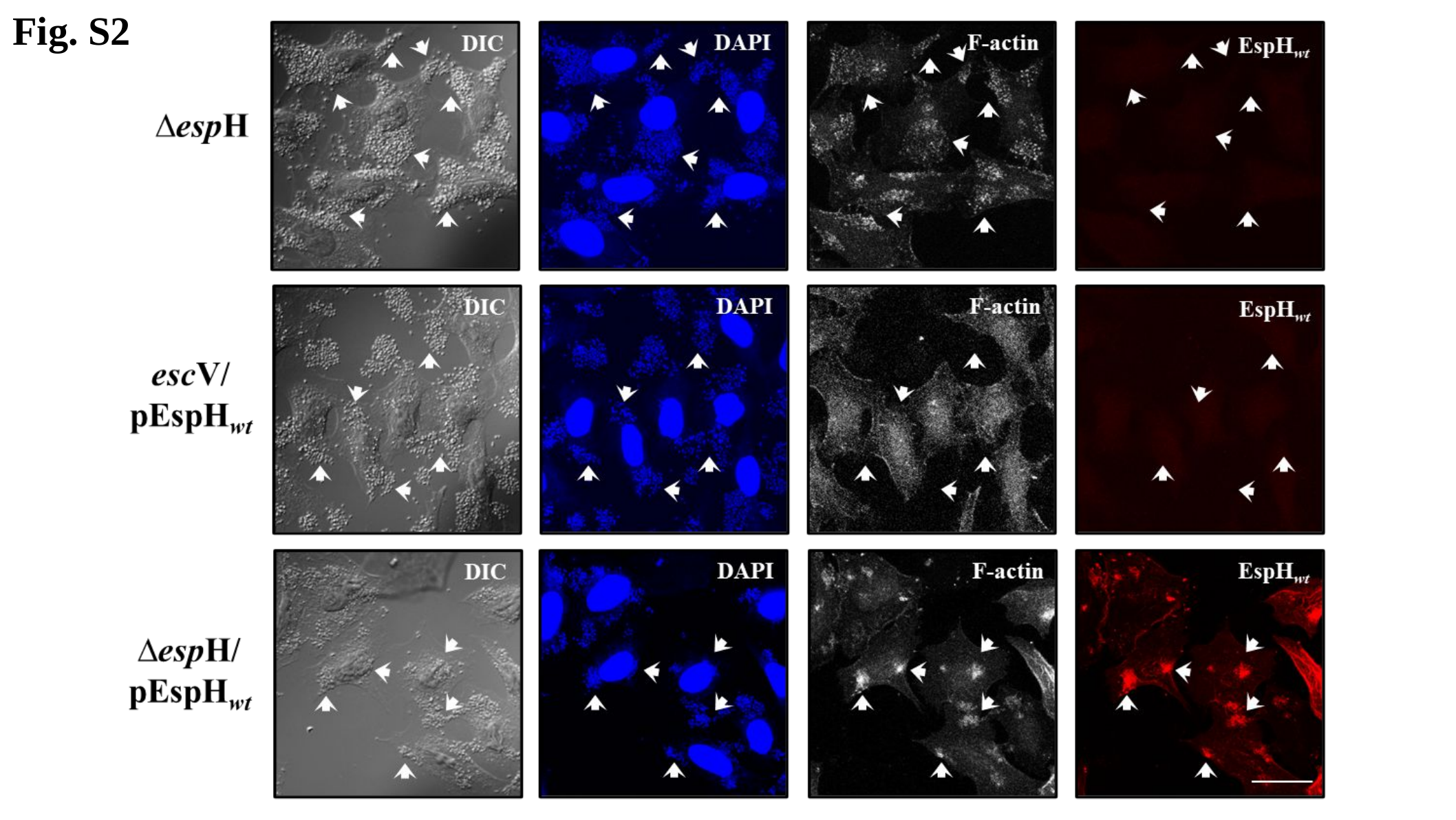

Fig. S2

## Slide 3
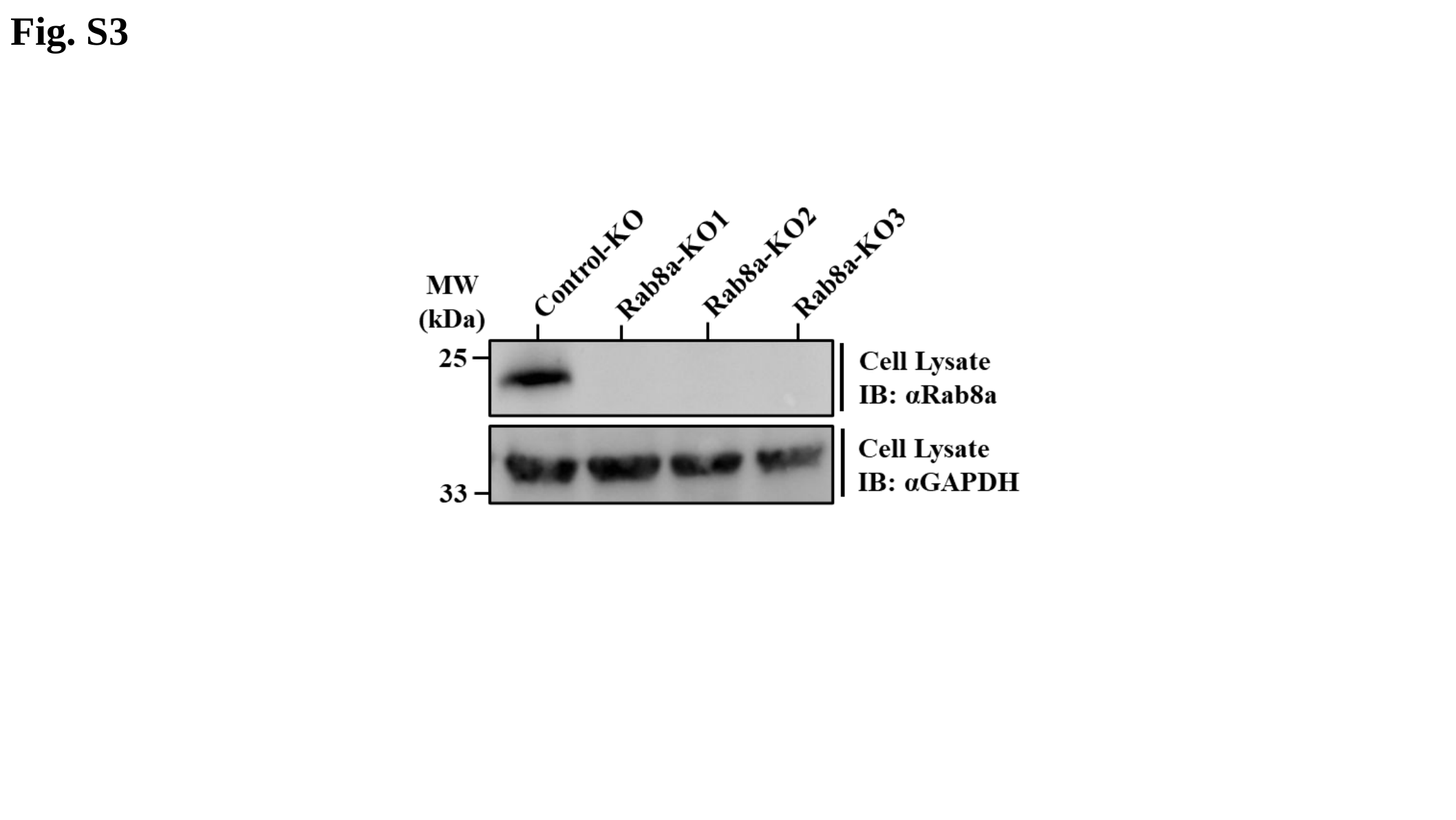

Fig. S3

## Slide 4
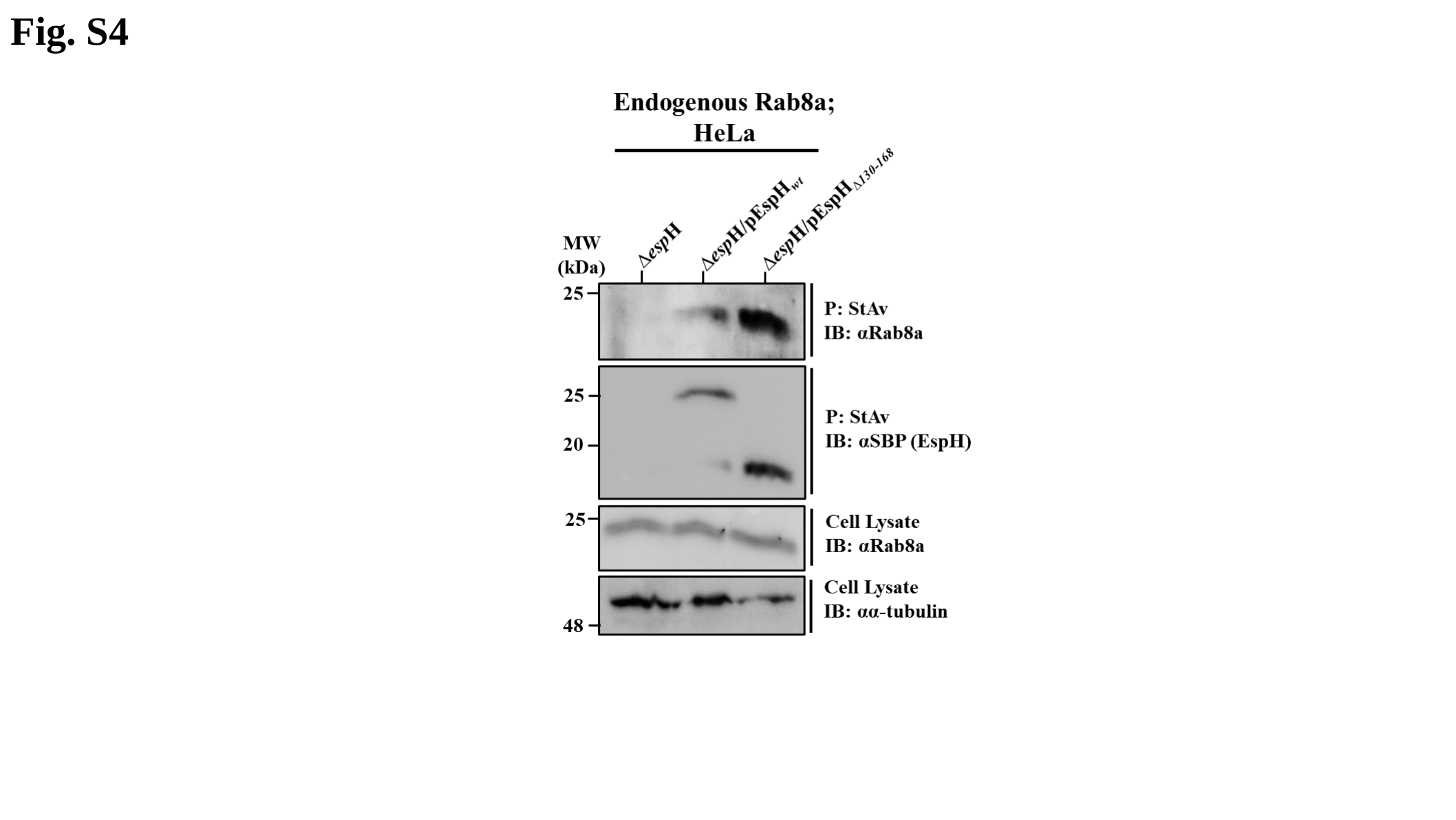

Fig. S4

## Slide 5
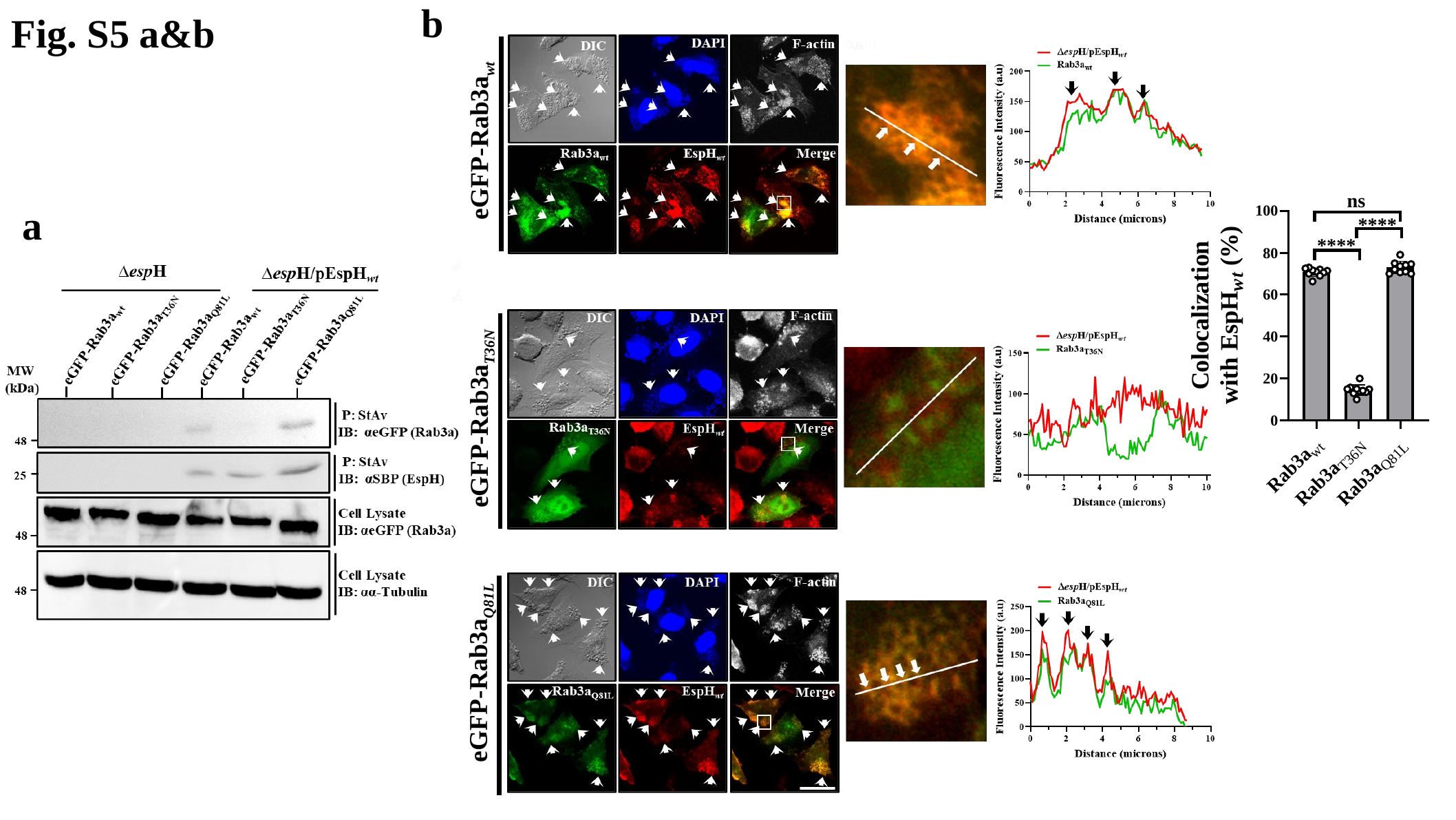

b
Fig. S5 a&b
eGFP-Rab3awt
a
eGFP-Rab3aT36N
eGFP-Rab3aQ81L

## Slide 6
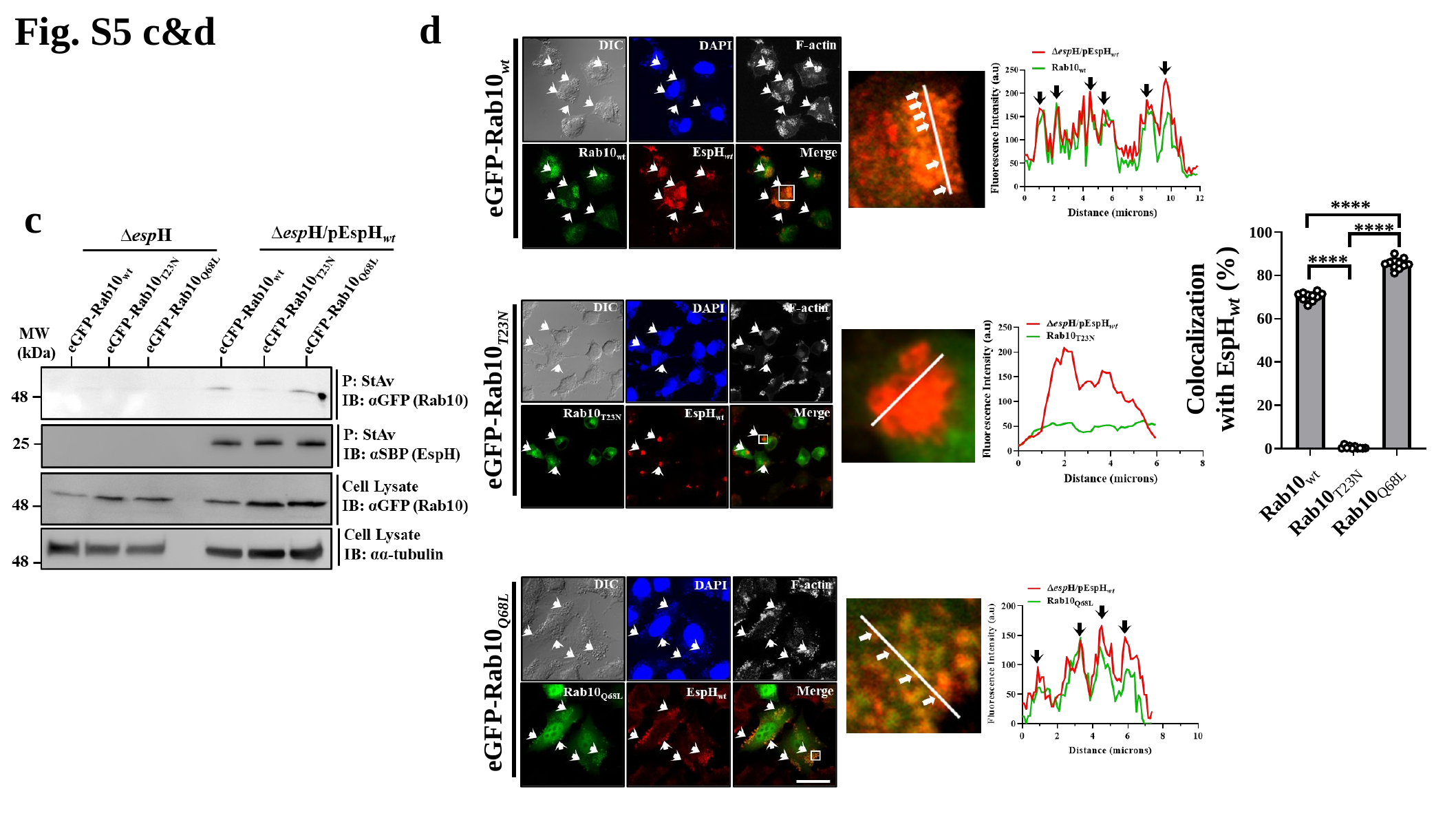

Fig. S5 c&d
d
eGFP-Rab10wt
c
eGFP-Rab10T23N
eGFP-Rab10Q68L

## Slide 7
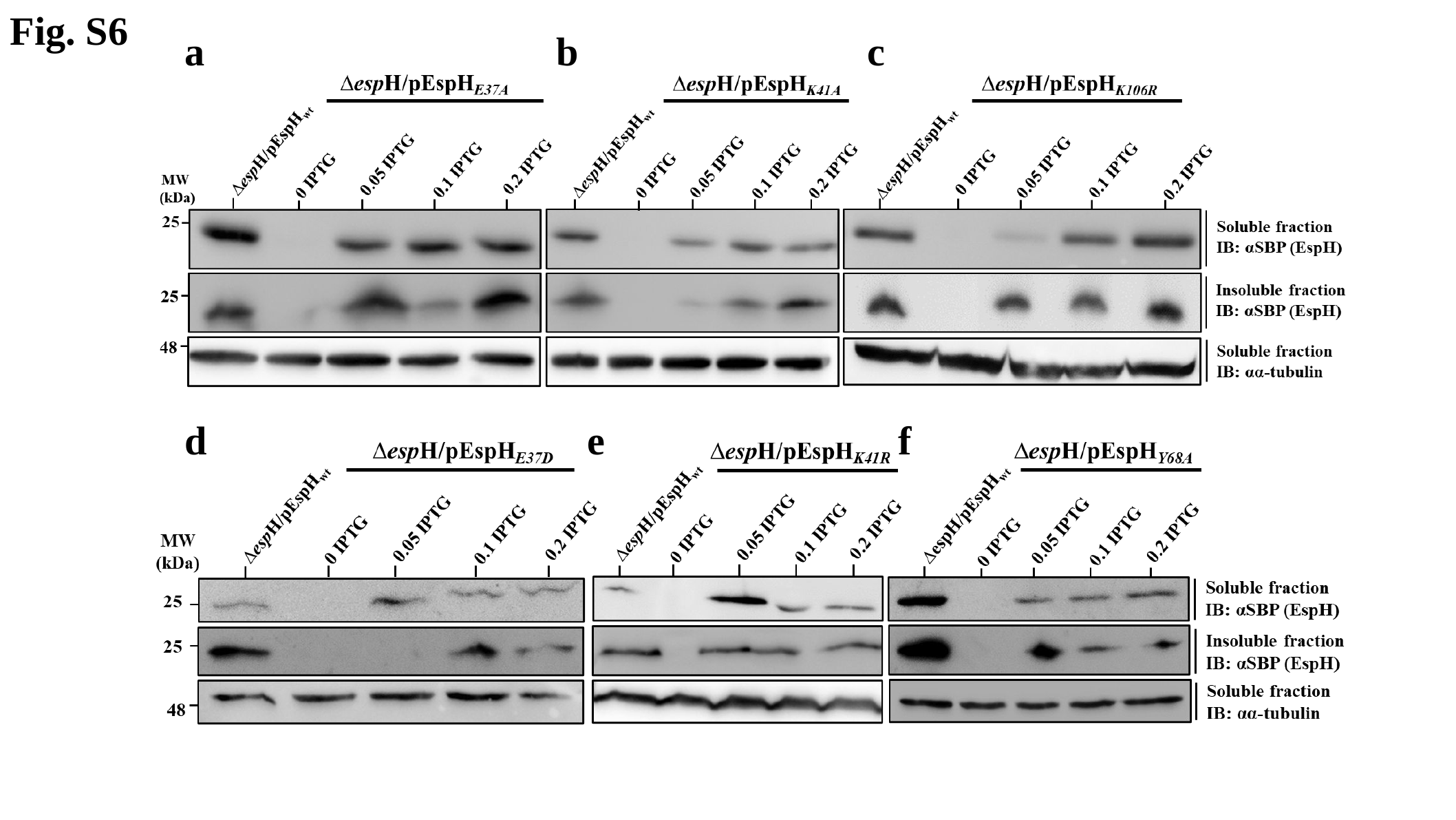

Fig. S6
c
b
a
f
e
d

## Slide 8
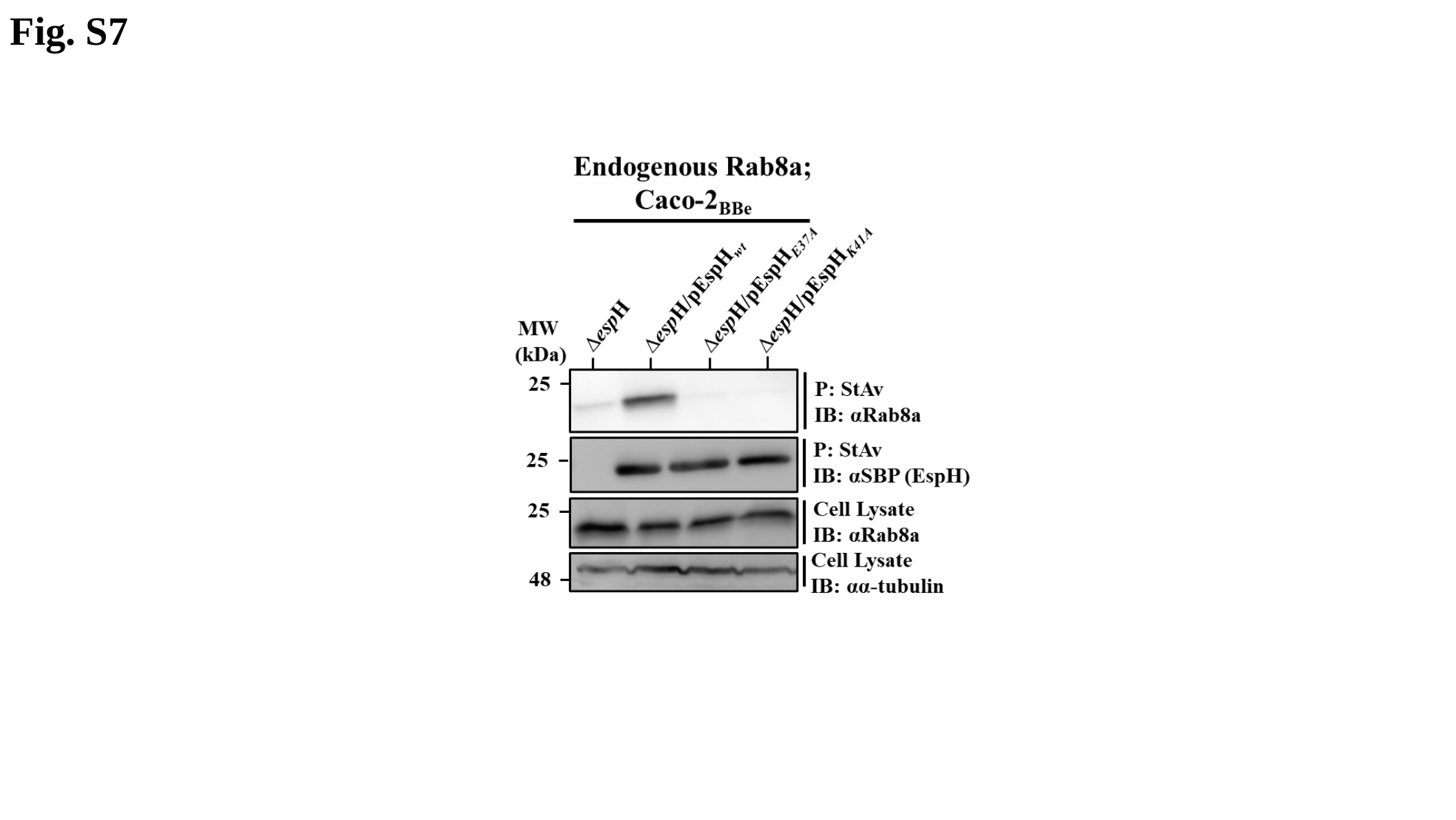

Fig. S7

## Slide 9
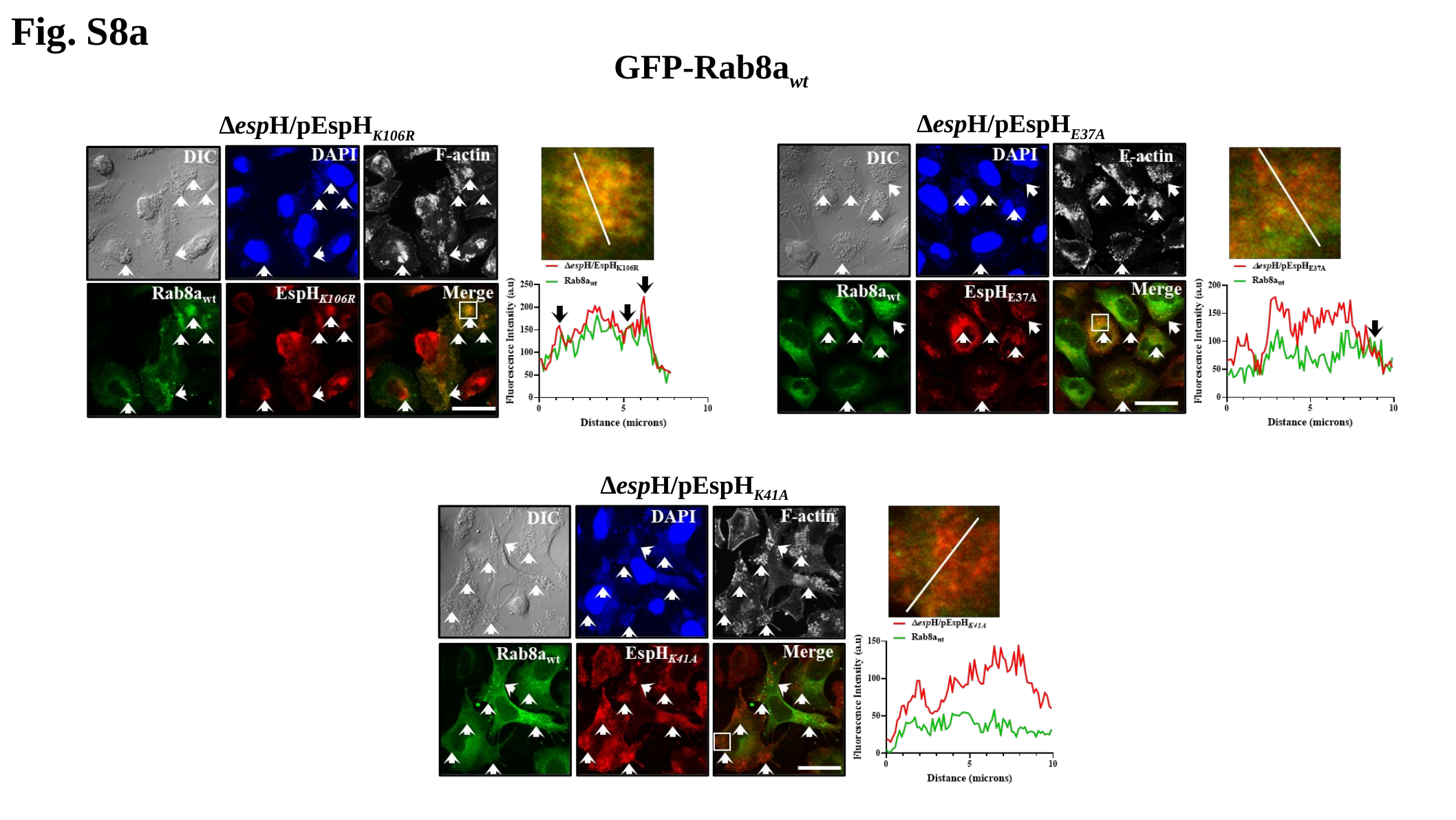

Fig. S8a
GFP-Rab8awt
∆espH/pEspHE37A
∆espH/pEspHK106R
∆espH/pEspHK41A

## Slide 10
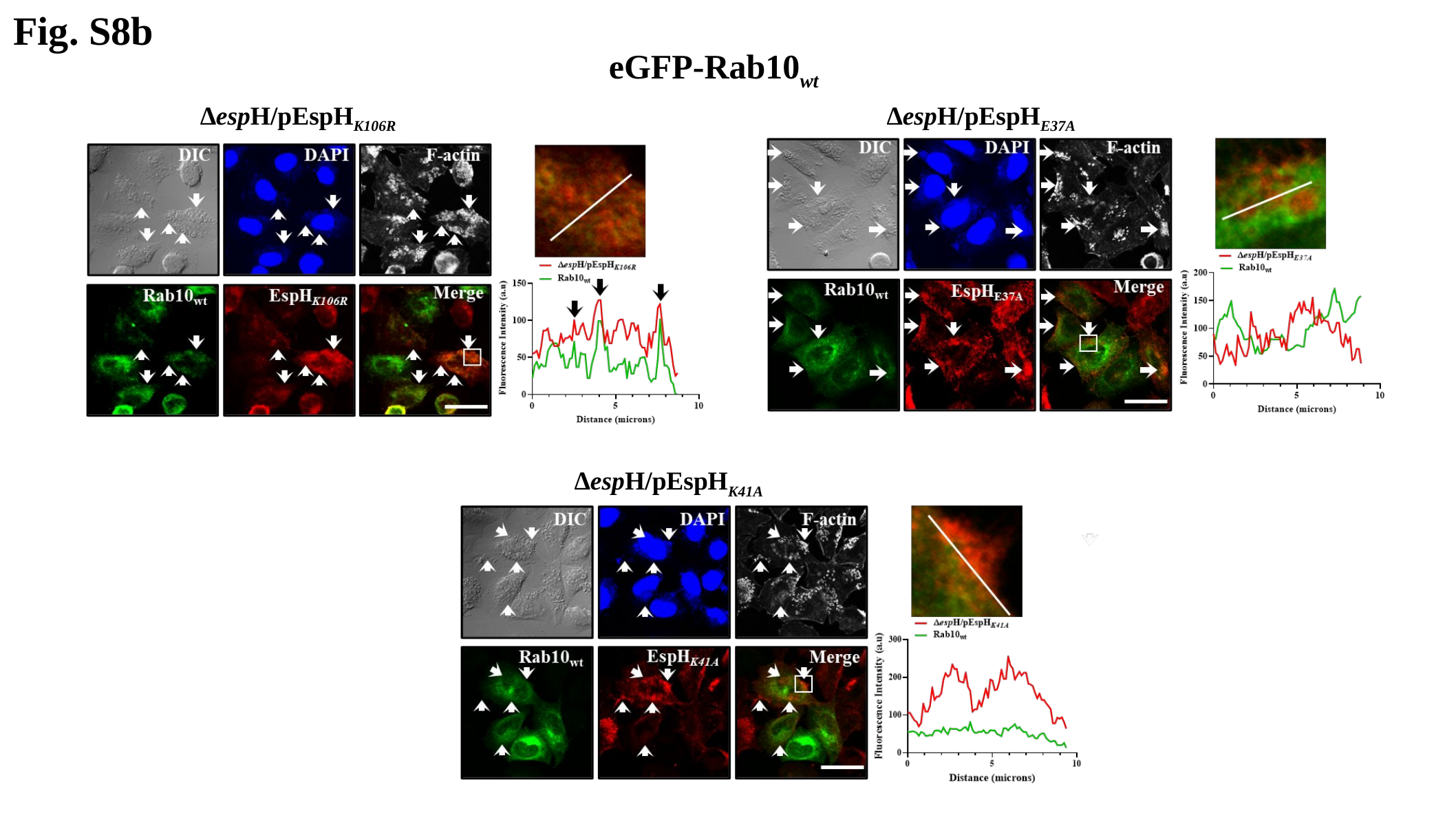

Fig. S8b
eGFP-Rab10wt
∆espH/pEspHK106R
∆espH/pEspHE37A
∆espH/pEspHK41A

## Slide 11
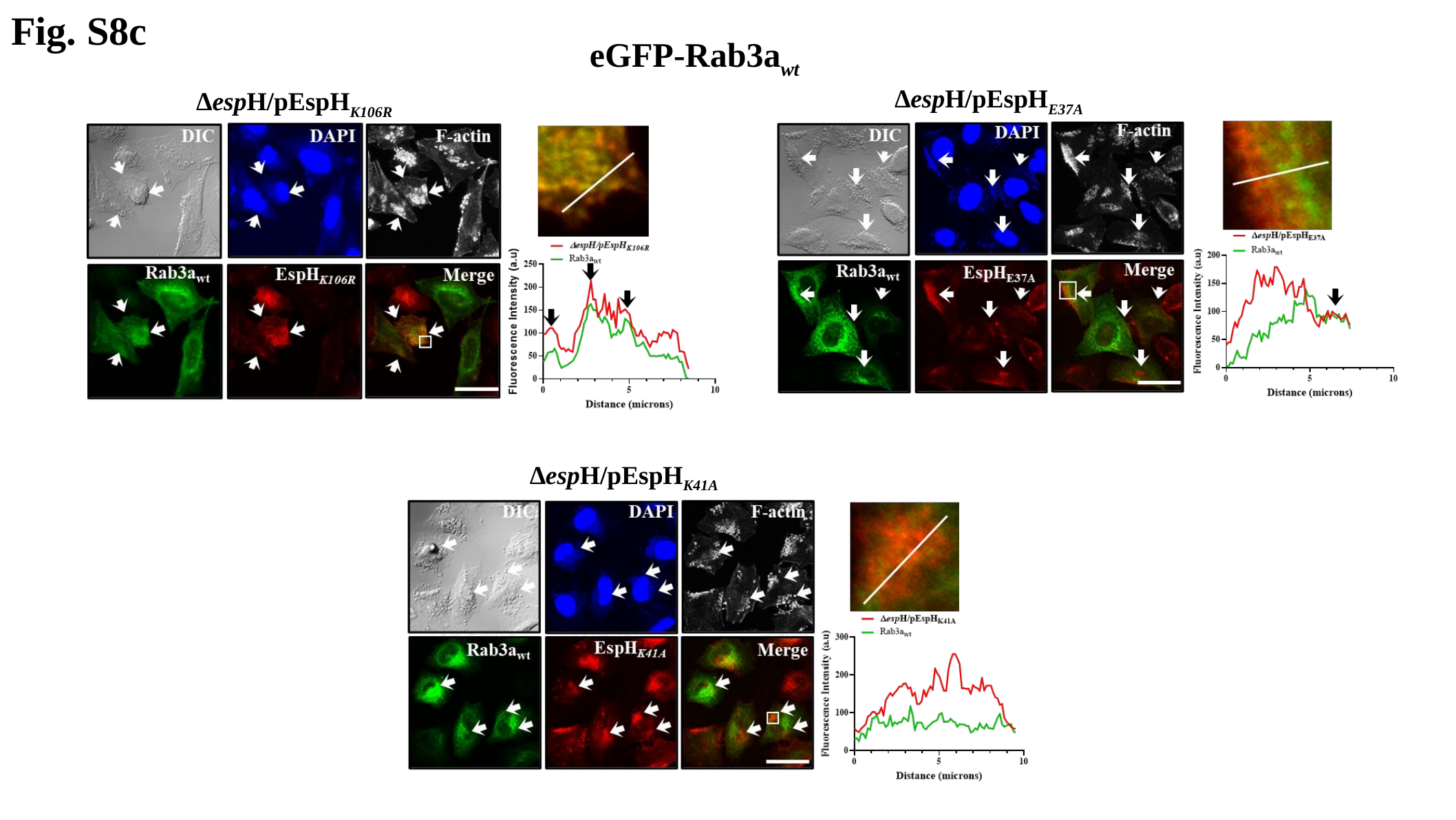

Fig. S8c
eGFP-Rab3awt
∆espH/pEspHE37A
∆espH/pEspHK106R
∆espH/pEspHK41A

## Slide 12
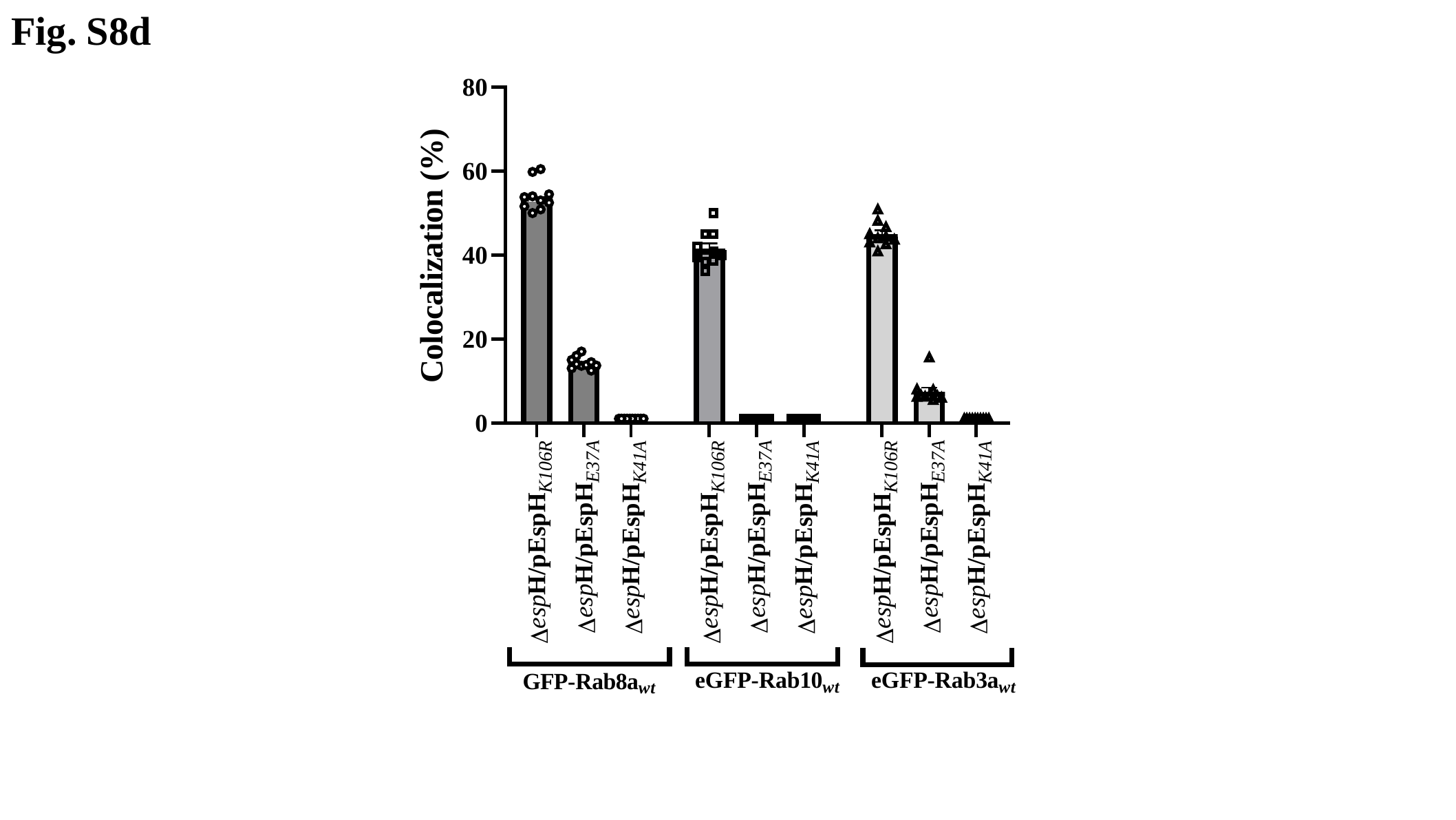

Fig. S8d

## Slide 13
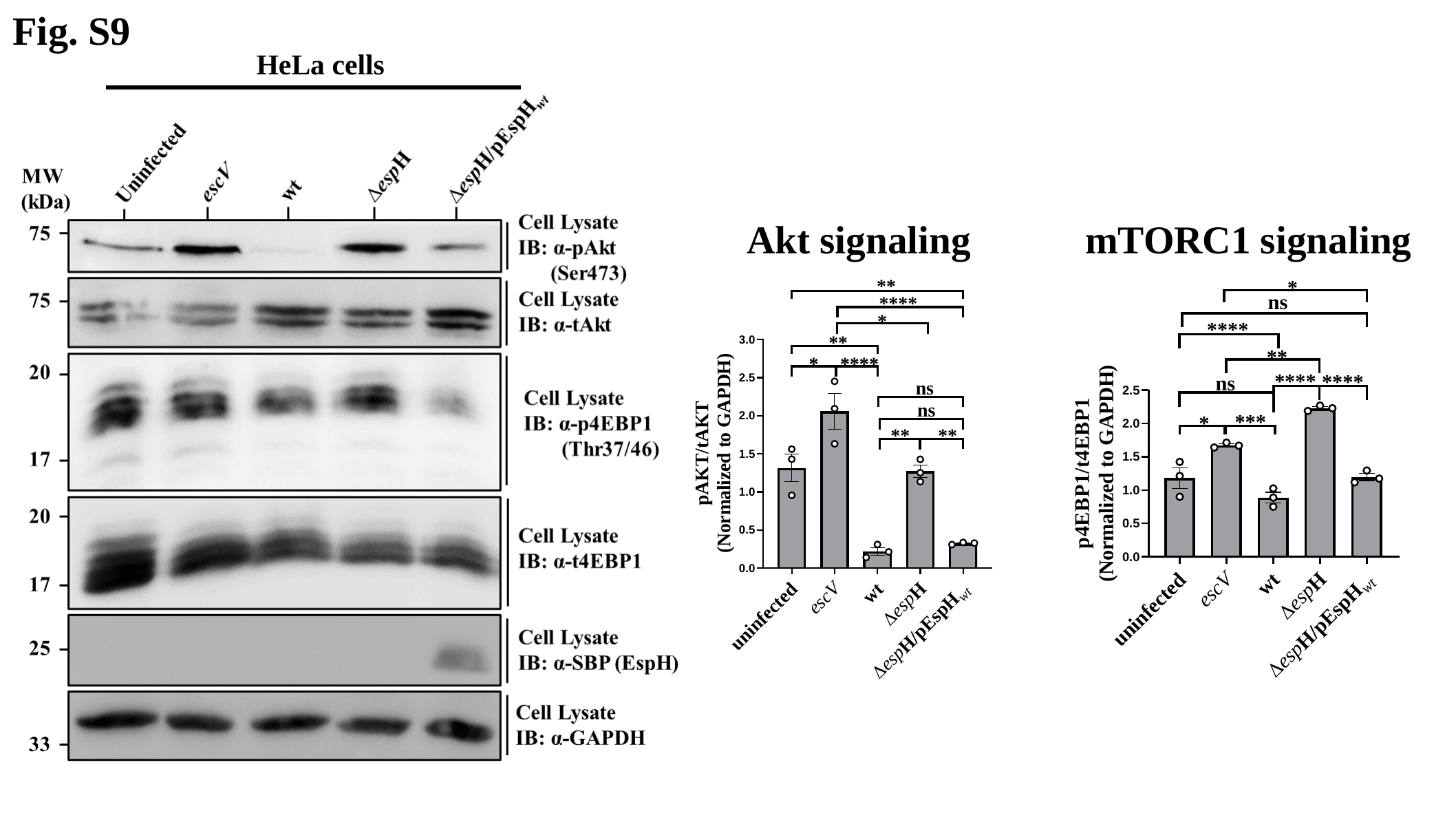

Fig. S9
 HeLa cells
mTORC1 signaling
Akt signaling

## Slide 14
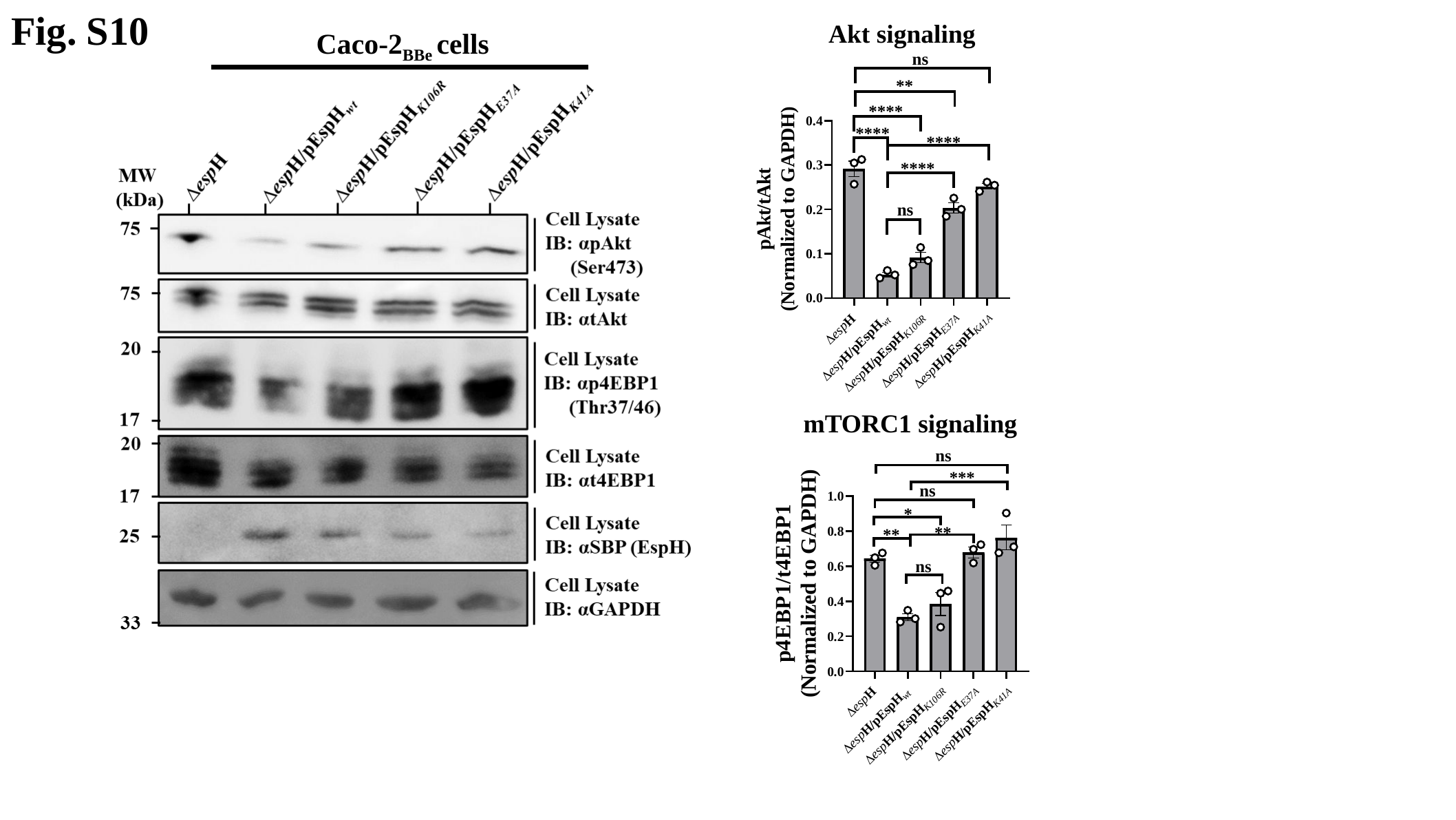

Fig. S10
Akt signaling
Caco-2BBe cells
mTORC1 signaling

## Slide 15
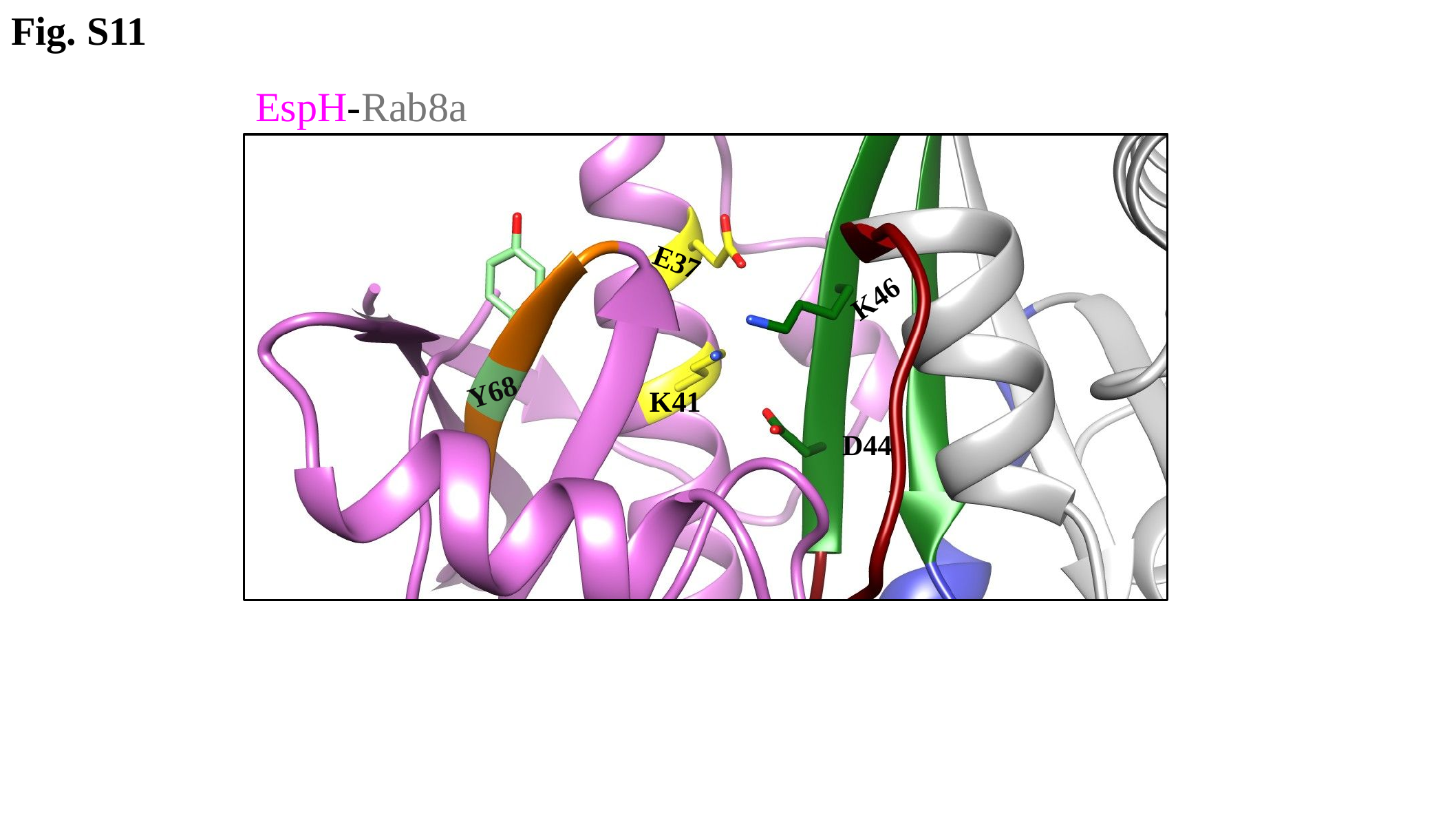

Fig. S11
EspH-Rab8a
E37
K46
Y68
K41
D44

## Slide 16
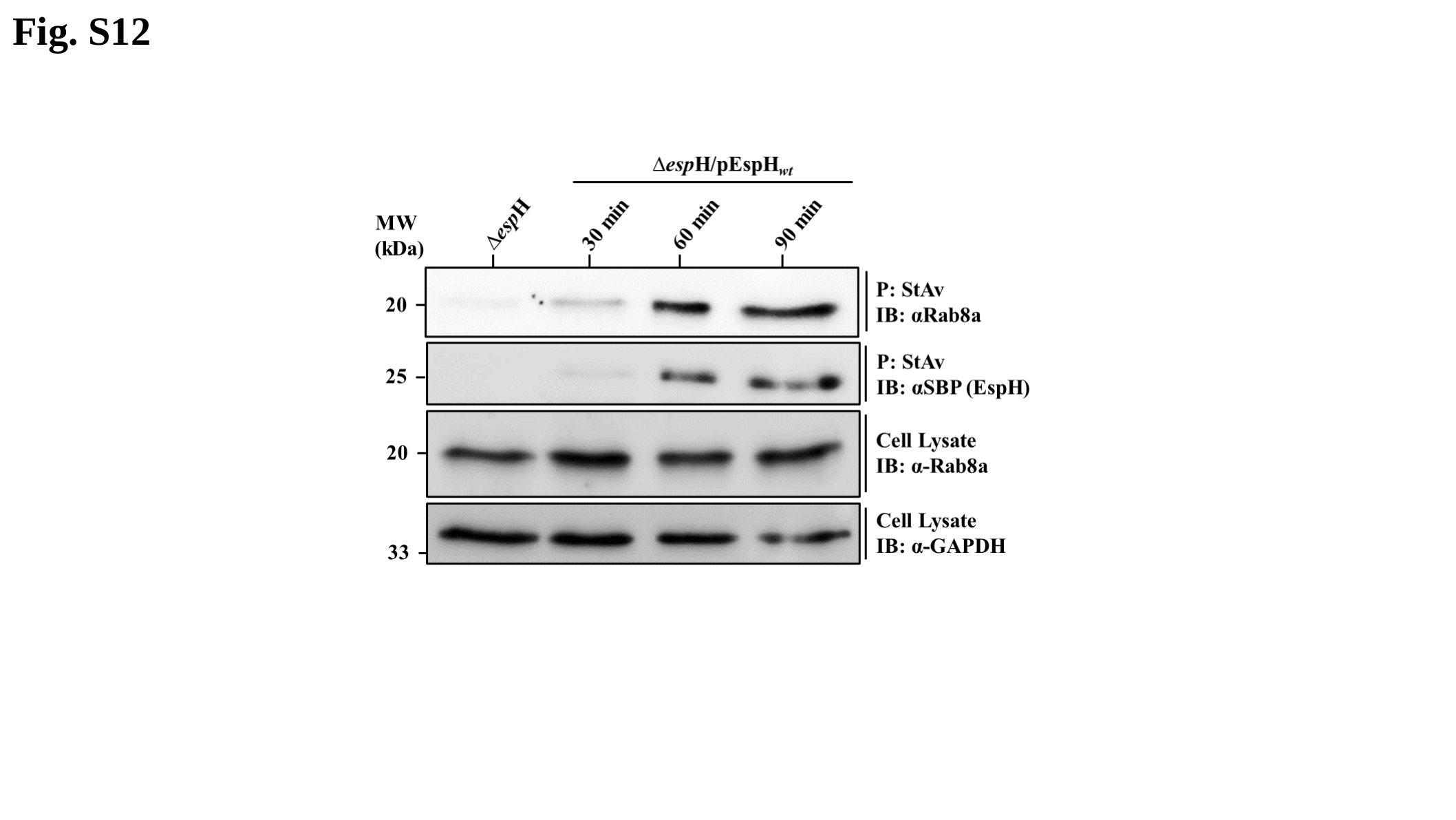

Fig. S12
