# Supplementary material for: EspH utilizes phosphoinositide and Rab binding domains to interact with plasma membrane infection sites and Rab GTPases*
Source: Gut Microbes. 2024 Sep 23;16(1):2400575. doi: 10.1080/19490976.2024.2400575 (PMC11421376; doi:10.1080/19490976.2024.2400575)
Supplement: Supplemental Material [file KGMI_A_2400575_SM0660.zip › Supplementary_Figure_legends_changes_after_proofs_.docx]

**Supplementary Figure legends**

**Fig. S1: Clustal aa sequence alignment of EspH effector proteins expressed by pathogenic *E. coli*.** The Genebank names of EspH are indicated on the left, and amino acid (aa) numbers are shown on the right. The corresponding pathogenic *E. coli* strains are as follows: ECSP_4679- O157:H7 str TW14359 (EHEC); ECO103_3614- O103:H2 str 12009 (VTEC); ECO26_5275- O26:H11 str 11368 (STEC); ROD_29891- *Citrobacter rodentium* ICC168; ECO111_3748- O111:H- str 11128 (EHEC); E2348C_3944- O127:H6 str E2348/69 (EPEC). The CLUSTAL O (1.2.4) multiple sequence alignment program (<https://doi.org/10.1093/nar/gkac240>) was used. * residues indicate positions having a single and fully conserved residue. : indicates conserved displaying strongly similar properties (score > 0.5 on the PAM 250 matrix). · indicates semi-conserved residues showing weakly similar properties (score ≤ 0.5 on the PAM 250 matrix). Blanks indicate non-conserved residues. The black vertical boxes indicate the residues mutated in this study (i.e., E35A/D, K41A/R, Y68A, and K106R). The yellow highlighted region indicates the common GKxYx_n_F PI-binding domain (PBD). The horizontal box indicates the C-terminal 38 aa segment ABR binding motif.

**Fig. S2: Translocated EspH is labeled specifically Anti-SBP antibodies.** HeLa cells were infected with the indicated EPEC strains for 90 min at 37 °C, fixed, permeabilized, and immunostained with anti-SBP antibodies to visualize EspH. Cells were stained with DAPI (to visualize cell nuclei and bacterial microcolonies) and Phalloidin CF-647 (F-actin) and imaged by confocal microscopy. Representative images from three independent experiments are shown. Arrows point towards infecting EPEC microcolonies.

**Fig. S3: Analysis of CRISPR/Cas9 Rab8a KO cells by IB.** The Control-KO and 4 single Rab8a-KO clone cells were lysed and analyzed by IB for the presence of endogenous Rab8a using anti-Rab8a antibodies. Anti-GAPDH antibodies were used to evaluate protein loading. Rab8a-KO1 was used for further experiments.

**Fig. S4: Rab8a is co-precipitated with translocated EspH*_∆130-168_*.** Experiments were performed as described in **Fig. 1** and Materials and Methods. A representative gel from three independent experiments is shown.

**Fig. S5: Translocated EspH*_wt_* interacts with active Rab3a (a and b) and Rab10 (c and d).** Experiments were performed as described in Materials and Methods and **Fig. 2**. Rab3a_T36N_, Rab3a_Q81L_, Rab10_T23N_, and Rab10_Q68L_ represent the dominant negative (GDP-bound) and constitutively active (GTP-bound) Rab forms, respectively. Representative gels (panel a), confocal images, and colocalization analyses (panel b) from three independent experiments are shown.

**Fig. S6:** **Translocation of EspH mutants.** HeLa cells were infected for 90 min at 37 °C with EPEC-Δ*espH*/pEspH*_E37A_* **(a)**, EPEC-Δ*espH*/pEspH*_K41A_* **(b)**, EPEC-Δ*espH*/pEspH*_K106R_* **(c)**, EPEC-Δ*espH*/pEspH*_E37D_* **(d)**, EPEC-Δ*espH*/pEspH*_K41R_* **(e)***_,_* or EPEC-Δ*espH*/pEspH*_Y68A_* **(f)**. The different concentrations of IPTG were used to induce effector protein expression levels. The effector translocation assay was applied, as described in Materials and Methods. EspH was detected using anti-SBP antibodies. Anti-α-tubulin (αα-tubulin) antibodies were used to evaluate protein loading.

**Fig. S7: The Rab binding residues E37 and K41 of EspH are critical for binding endogenous Rab8a in Caco-2_BBe_ cells.** Caco-2_BBe_ cells were infected with indicated EPEC strains for 90 min at 37 °C, and a co-precipitation experiment was carried out, as described in Materials and Methods and **Fig. 4**.

**Fig. S8: The Rab binding residues in EspH are critical for recruiting Rabs to infection sites.** HeLa cells expressing GFP-Rab8a*_wt_* (**a**), eGFP-Rab10*_wt_* (**b**), or eGFP-Rab3a*_wt_* (**c**) were infected with the indicated EPEC strains for 90 min at 37 °C. Cells were subjected to EspH immunostaining using anti-SBP antibodies, DAPI (to visualize host nuclei and bacterial microcolonies (pointed with arrowheads), Phalloidin CF-647 (to visualize F-actin), and confocal imaging followed by colocalization analysis, performed as described in **Fig. 2B** (**d**). Results are mean ±SE.

**Fig. S9: Translocated EspH inhibits the Akt/mTORC1 signaling in HeLa cells.** HeLa cells were infected or not (uninfected) with the indicated EPEC strains for 90 min at 37 °C, and the effect on Akt and mTORC1 signaling was measured as described in **Fig. 5** and Materials and Methods. Results are mean ± SE.

**Fig. S10: The Rab binding residues in EspH are critical for exerting Akt/mTORC1 signaling in Caco-2_BBe_ cells.** Caco-2_BBe_ cells were infected with indicated EPEC strains for 90 min at 37 °C, and the effects on Akt and mTORC1 signaling were assessed, as described in **Fig. 5** and Materials and Methods. Results are mean ± SE.

**Fig. S11: AlphaFold predicted structure of EspH-Rab8a shows that the Y68 residue of the EspH PBD is distinct from the Rab binding site.** The complex of EspH-Rab8a was modeled using AlphaFold-Multimer-v2.0 (see also **Fig. 3**). The EspH (pink) and the Rab GTPase (gray) structures are depicted. The switch I, interswitch, and switch II Rab domains are shown in maroon, dark green, and navy blue, respectively. The interface area shows the predicted interacting E37 and K41 of EspH (yellow) and K46 and D44 of Rab8a interswitch region (green). The PBD of EspH (orange) is shown with Y68 (light green).

**Fig. S12: Rab8a interacts with translocated EspH at early (30 min) and late (90 min) phases.** HeLa cells were infected with the indicated EPEC strains at different timepoints (30 min, 60 min, and 90 min), and a co-precipitation experiment was carried out, as described in Materials and Methods and **Fig. 1**.
